# Short and long-term acceptability and efficacy of extended-release cornstarch in the hepatic glycogen storage diseases: results from the Glyde study

**DOI:** 10.1186/s13023-024-03274-y

**Published:** 2024-07-09

**Authors:** DA Weinstein, RJ Jackson, EA Brennan, M Williams, JE Davison, F de Boer, TGJ Derks, C Ellerton, B Faragher, J Gribben, P Labrune, KM McKittrick, E Murphy, KM Ross, U Steuerwald, C Voillot, AJM Woodward, HR Mundy

**Affiliations:** 1 Glycogen Storage Disease Program, Connecticut Childrens Medical Center, Hartford, USA; 2 School of Medicine, Department of Pediatrics, University of Connecticut, Farmington, CT USA; 3 Liverpool Clinical Trials Centre, University of LiverpoolUK, Liverpool, UK; 4 Vitaflo International Ltd, 182 Sefton Street, Liverpool, UK; 5 Metabolic Medicine, Great Ormond Street Hospital, London, UK; 6 Division of Metabolic Diseases, Beatrix Children’s Hospital, University Medical Center Groningen, Groningen, The Netherlands; 7 Charles Dent Metabolic Unit, National Hospital for Neurology and Neurosurgery, Queen Square, London, UK; 8 Liverpool School of Tropical Medicine, Pembroke Place, Liverpool, UK; 9 Evelina London Childrens Hospital, Westminster Bridge Road, London, UK; 10 Centre de Référence des Maladies héréditaires du Métabolisme Hépatique, APHP, Hôpitaux Universitaires Paris-Saclay, Hôpital Antoine Béclère, Clamart, and Paris-Saclay University, Paris, France; 11 National Hospital of the Faroe Islands, Medical Center, Tórshavn, Faroe Islands

## Abstract

**Background:**

Hypoglycaemia is the primary manifestation of all the hepatic types of glycogen storage disease (GSD). In 2008, Glycosade^®^, an extended-release waxy maize cornstarch, was reported as an alternative to uncooked cornstarch (UCCS) which could prolong the duration of fasting in the GSD population. To date, there has been minimal published experience in (a) young children, (b) the ketotic forms of GSD, and (c) with daytime dosing. The Glyde study was created as a prospective, global initiative to test the efficacy and tolerance of Glycosade use across a broader and more diverse population.

**Methods:**

A randomised double-blind cross-over fasting study assessing the tolerance and efficacy of Glycosade compared with cornstarch was performed across disease types and ages. Participants and clinicians chose the product deemed superior, whilst still blinded. Participants were followed for 2 years to assess long-term metabolic control, growth, and quality of life.

**Results:**

Sixty-one participants (age 2–62 years; 59% female) were enrolled, and 58 participants completed the fasting studies (28 GSD I; 30 GSD III, VI, IX). Glycosade improved duration of fasting in GSD I and duration of fasting without ketosis in the ketotic forms. Chronic Glycosade use was chosen by 69% of participants. Those treated with Glycosade for the 2-year chronic phase used fewer doses of therapy while markers of metabolic control remained stable.

**Conclusion:**

The Glyde study is the first multi-centre international trial demonstrating the efficacy and tolerance of Glycosade in a large cohort of hepatic GSD patients across a diverse international population. The ability to use fewer doses of therapy per day and avoidance of overnight therapy may improve compliance, safety, and quality of life without sacrificing metabolic control.

**Supplementary Information:**

The online version contains supplementary material available at 10.1186/s13023-024-03274-y.

## Introduction

The hepatic glycogen storage diseases (GSDs) are a group of inherited disorders characterised by the abnormal storage or release of glycogen [1]. The inability to release glycogen as glucose during periods of fasting results in marked hypoglycaemia. In glycogen storage disease type I (GSD I), both glycogenolysis and gluconeogenesis are impaired. Consequently, all endogenous glucose production is compromised resulting in severe hypoglycaemia, and the shunting of glucose-6-phosphate into alternative pathways results in the accumulation of uric acid, triglycerides, and lactate. In the other hepatic forms of GSD (GSD 0, III, VI, and IX), gluconeogenesis and fatty acid oxidation are preserved resulting in milder hypoglycaemia and slightly longer fasting tolerance; increased beta-oxidation, however, results in ketosis (Weinstein, Steuerwald, De Souza, & Derks, [2]).

While there is a spectrum of severity across the different hepatic forms of glycogen storage disease, hypoglycaemia is the primary manifestation of them all. The general aims of dietary management are to achieve normoglycaemia, normalise secondary metabolic perturbations, and prevent long-term chronic complications, while preserving quality of live. Prior to the description of continuous glucose therapy for treatment of GSD I, most patients with GSD did not survive [3]. With the introduction of uncooked cornstarch (UCCS) therapy in the early 1980s, the mortality and morbidity for children and adults with GSD improved, but dosing every 3–5 h was required to achieve optimal metabolic control (Chen, Cornblath, & Sidbury, [4]; Lee, Dixon, & Leonard, [5]; Weinstein, Wolfsdorf, [6]). In 2008, an extended-release waxy maize cornstarch was reported as an alternative to UCCS which could prolong the duration of fasting in the GSD Ia population [7, 8] and allow many patients to sleep through the night without awakening for therapy. In 2009, the product branded as Glycosade^®^ (Vitaflo International Ltd, Liverpool, England) was approved as a food for medical purposes for the dietary management of GSD in the United Kingdom and was launched as a medical food in the USA in 2012.

Despite extensive use over the past 15 years, there is limited published experience regarding the use of Glycosade in all ages and hepatic GSD types. While the published experience has documented improved safety through avoidance of overnight cornstarch therapy and improved quality of life, many questions have not been addressed to date [9–14]. The published experience predominantly focused on overnight use of Glycosade, and long-term use during the day has not been addressed. The literature has focused primarily on use in the GSD Ia population, and there has been minimal published experience in young children since Glycosade is recommended for use in those over two years of age except for the USA where the age indication is over five years of age.

The Glyde study was created as a prospective, global initiative to test the efficacy and tolerance of Glycosade use across a broader and more diverse population. Prospective, cross-over, double-blind fasting challenges were performed comparing UCCS and Glycosade at six centres in four countries to obtain efficacy and tolerance data within the common hepatic forms of GSD (GSD I, III, VI, and IX). Following the challenges, the clinicians and participants chose the product deemed to be superior without knowledge of the identity of the test product, and longer-term clinical data were obtained to assess the impact of the therapy on metabolic control, growth, dietary intake, and quality of life. The results from the international, prospective study are now presented expanding the short and long-term experience across the age spectrum and disease types.

## Methods

### Study design

Glyde was designed as a two-part study to assess the short and long-term use of Glycosade (Supplementary Figs. 1 & 2). The first part consisted of a randomised double-blind, cross-over fasting study assessing the tolerance and efficacy of Glycosade compared with UCCS across disease types and ages. Participants were randomised using equal allocation to receive either Glycosade followed by UCCS or the alternative order. The same UCCS was used across all sites to eliminate any potential differences due to geography. Studies were performed a minimum of two weeks apart, after which participants and clinicians chose the product deemed superior for long-term use, whilst still blinded. Biochemical parameters and participant acceptability of the starches were used to inform this decision. Participants were then followed with annual visits to the site for two years to assess daily glucose profiles through hourly assessment at the research centre, laboratory studies l, growth, and quality of life which defined the second part of the study. While laboratory studies to assess markers of metabolic control were obtained during the study visits, additional studies could be obtained as clinically indicated. Imaging and renal studies were not obtained as part of the investigations, but these data were collected as per clinical standards during visits four and five. During this period, starch doses continued to be titrated as dictated by standards of care by the primary team. Continuous glucose monitoring (CGM) was not performed as part of the research trial since the technology had not been universally accepted when the trial commenced. Validation studies of CGM devices were performed as separate investigations obtained during the fasting challenges and study visits, and these results were published as part of the manuscript published by Peeks et al. [15]. Dietary intake data was also collected at baseline, visit four and five however due to the volume of data this will be published separately.

### Participants

Participants were eligible for the study based on a diagnosis of Glycogen Storage Disease Types I, III, VI or IX under the care of a metabolic centre meeting the following inclusion criteria: diagnosed by genetic analysis or enzymology study, aged two years or older (five years or older in the USA), and established on full intake of UCCS for at least 6 months. Exclusion criteria included women who were pregnant or breastfeeding at the start of the study or planning to become pregnant during the study. Participants were included in the study for a period of two years encompassing an initial cross over study and longer-term clinical follow-up of the chosen dietary product (Supplementary Fig. 1). Baseline pharmacologic therapies for GSD I are summarized in Table 1. Participants with GSD III, VI and IX were not prescribed any medications which could impact metabolic control. The study was sponsored and funded by Vitaflo (International) Ltd. Ethical approval at all centres was obtained prior to study opening. In the United Kingdom (UK) ethical approval was received from the NRES Committee London- Queen Square. Ref: 15/LO/0685, IRAS project ID 148,762. In France a favourable opinion was received from Comite de Protection des Personnes (CPP) Ile de France XI, CPP ref: 16,012. Ethical approval was received from the Institutional Review Board (IRB) of Connecticut Children’s Medical Centre, CCMC IRB# 16–101. In the Netherlands a favourable opinion was received from Universitair Medisch Ethische Toetsingscommissie: University Medical Centre Groningen (METc UMCG). The study opened in six international sites and was registered at http://clinicaltrial.gov (NCT02318966) on 12th December 2014.

**Table 1:** Patient demographics

		Evaluable patients only
**All patients**		
Date of study entry		23/02/16 : 25/06/18
Age (years)	median [range]	12.0 [ 2.0 : 62.8]
Age Category: – Child (< 14 years)	n (%)	24 (41)
- Adult (> 14 years)	n (%)	34 (59)
Sex – Female	n (%)	24 (41)
Male	n (%)	34 (59)
Type of GSD: Ia	n (%)	27(46)
Ib	n (%)	1 (2)
IIIa / IIIb	n (%)	15 (26)
IX	n (%)	14 (24)
VI	n (%)	1 (2)
Baseline starch: UCCS	n (%)	34 (59)
Glycosade	n (%)	6 (10)
Combination of both	n (%)	18 (31)
Baseline Medications: GSD I		
Uric Acid lowering	n (%)	12 (44)
Kidney Stone Prophylaxis	n (%)	4 (15)
Lipid lowering medications	n (%)	2 (7%)
Hypertension medications	n (%)	6 (22)

### Intervention

The study intervention was Glycosade, a food for special medical purposes (as defined by Directive 1999/21/EC) for the dietary management of glycogen storage disease. The comparator intervention was UCCS. For the fasting challenges, the starches were dosed by providing 2 g carbohydrate per kilogram ideal body weight up to a maximum of 100 g of carbohydrate. The dosing was performed to standardise the carbohydrate load administered since the products have different percentages of carbohydrate by weight. The protocol was standardised to minimize variability in diet and activity prior and during the blinded fasting challenges, and cornstarch doses were adjusted prior to the studies to ensure that the fasting protocols were performed when therapy was due to be administered to minimize the impact of the baseline therapy (Supplementary Fig. 2). Blood glucose levels were measured in the research centre laboratory a minimum of hourly over a period of up to 12 h with an endpoint ≤ 3.6mmol/L. Lactate levels were also measured in the research centre laboratory for those with GSD I. β-hydroxybutyrate (BOHB) was assessed hourly during the fasting challenges only in participants with the ketotic forms of GSD (types III, VI, and IX) using the modified definition of the clinical endpoint (the development of significant ketosis as defined by BOHB ≥ 0.4 mmol/L) .

### Outcomes

The primary outcome of the first part of the study was the duration of normal blood glucose levels defined as a plasma glucose concentration ≥ 3.6mmol/L during the blinded fasting challenges. Secondary outcomes included blood lactate, beta-hydroxybutyrate (BOHB) levels, and 24-hour insulin profile. Further secondary outcomes associated with the longer-term follow-up beyond the initial cross over component of the study include the product choice for ongoing management, change in nutritional intake and biochemical parameters, chronic markers of metabolic control, and quality of life. Quality of life (QoL) was assessed using three instruments dependent upon age, Infant Toddler Quality of Life Questionnaire™ (ITQOL) for children less than 5 years, Child Health Questionnaire™ (CHQ-PF28) for children aged 6–18 years both from Quality Metric and 36-Item Short Form Survey (SFv36) (Rand Corporation) for adults. Side effects were assessed as any occurrences of adverse events (AEs) or Serious Adverse Events (SAEs) reported at any point during the study.

### Sample size

Sample size calculations were performed to detect a minimum one-hour difference in the period of normal blood glucose levels. Power calculations were based on an assumption that the standard deviation of the difference in the duration of normal blood glucose levels between UCCS and Glycosade would be 2.5 h [16]. Allowing for a 20% dropout rate, 64 participants were required for the study to achieve 80% power at a two-sided significance level of 5%.

### Randomisation and blinding

Participants were randomised under equal allocation to either: Group A (UCCS followed by Glycosade) or Group B (Glycosade followed by UCCS) using prepared lists generated on the principle of randomly permuted blocks. The randomisation schedule was stratified by site and prepared electronically by Sealed Envelope Ltd. The randomisation schedule was accessible online and solely by the study statistician and sponsor if the code break was required. Allocation concealment was managed locally by site with study product prepared by an unblinded independent clinical team member who was not affiliated with the trial.

### Statistical methods

Continuous data were summarised as median and interquartile range (IQR) and categorical data summarised as frequencies of counts with associated percentages. Analysis of the primary outcome was performed using a time-to-event approach, measuring the time until a participant recorded a blood glucose level ≤ 3.6mmol/L. Results are presented in terms of a hazard ratio (HR) (95% Confidence interval (CI)). Modelling was performed using Cox Proportional hazards modelling which adjusts for potential period effects. Analysis of BOHB was performed using the same methods as for the primary endpoint. Analyses of insulin and lactate were performed using regression techniques including terms to account for randomised groups and potential period effects with the impact of starch product assessed via inspection of a treatment-by-time interaction effect. Analyses of longitudinal outcomes were performed using Analysis of Covariance (ANCOVA) techniques, analyzing follow-up data whilst including data as adjusting covariates. All analyses were performed using SAS (Version 9) or R (Version 4). A *p*-value of 0.05 was used to define statistical significance throughout.

## Results

**Fig. 1:**
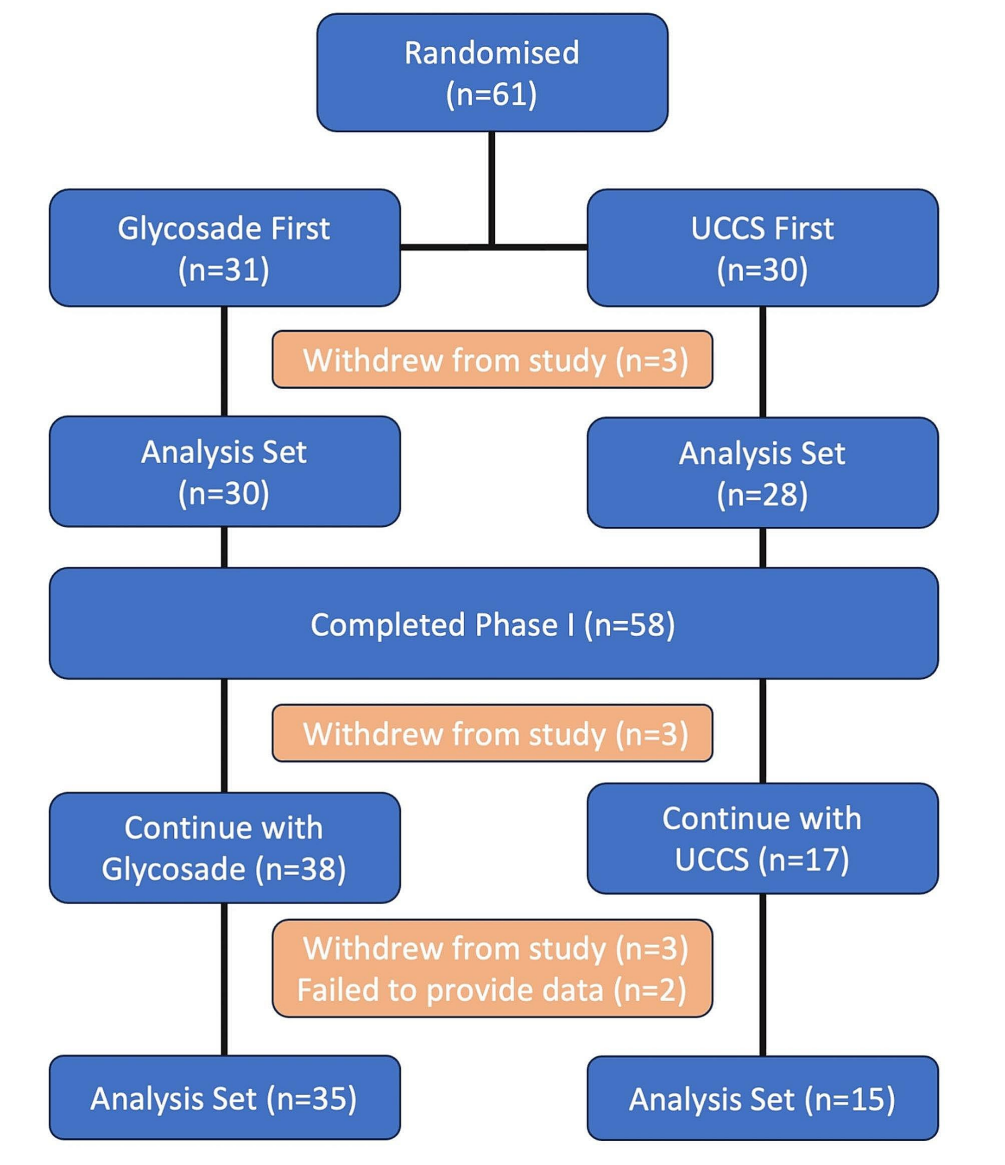
Consort Diagram

Sixty-one participants were recruited into the study between 23 February 2016 and 25 June 2018. Nine participants withdrew from study; three participants withdrew prior to completion of the fasting challenges due to difficulties with enteral administration of the test product or inability for intravenous access to be obtained. An additional six participants withdrew during the follow-up phase of the study; only one of these was deemed to be due to a starch treatment related AE (diarrhoea) [Fig. 1; Supplementary Table 1]. These participants were included in the analysis of the fasting challenges, but they were excluded from the long-term follow-up analysis due to lack of sufficient data to be included in the analysis set.

Of these 58 participants included in the statistical analyses, 30 (52%) were randomised into group 1 (Glycosade followed by UCCS) and 28 (48%) were randomised into group 2 (UCCS followed by Glycosade). The population had a median (range) age of 12.0 years (2.0–62 years) and were 59% female (34/58). Twenty-eight (48%) participants had a GSD Type I (27 with GSD Ia and one with GSD Ib) and 30 (52%) had GSD type III, VI or IX. Full details on participant demographics and baseline treatment strategy are included in Table 1. Genotyping confirmed the diagnosis for all the GSD I participants and for all but two of the ketotic GSD participants; those patients were diagnosed clinically and confirmed by enzyme assay (Supplementary Table 10).

### Blood glucose concentrations

Individual profile plots of blood glucose levels for participants on Glycosade and UCCS are included in Supplementary Figs. 4 & 5. Normal blood glucose levels were maintained for a significantly longer median time on Glycosade ([8.5 (6.5, > 12) hours]) than on UCCS ([7.5 (6.0, > 12) hours; HR (95% CI): 0.785 (0.624,0.988); *p*-value = 0.039]. Although a statistically significant difference was observed when all patients were considered together, this difference appeared to be confined primarily to the GSD I participants, particularly in those able to maintain normal blood glucose levels for 6 h or longer. Mean blood glucose was also maintained at a higher level by Glycosade compared to UCCS towards the end of the test, after most GSD I participants had ceased to maintain a normal glucose level (Supplementary Fig. 6b).

The ketotic GSD participants did not have blood glucose levels fall below 3.6 mmol/L, however mean blood glucose levels were also maintained at a higher level by Glycosade compared to UCCS towards the end of the starch load. Time to ketosis is a more sensitive marker of outcome for this group which is discussed below.

**Fig. 2:**
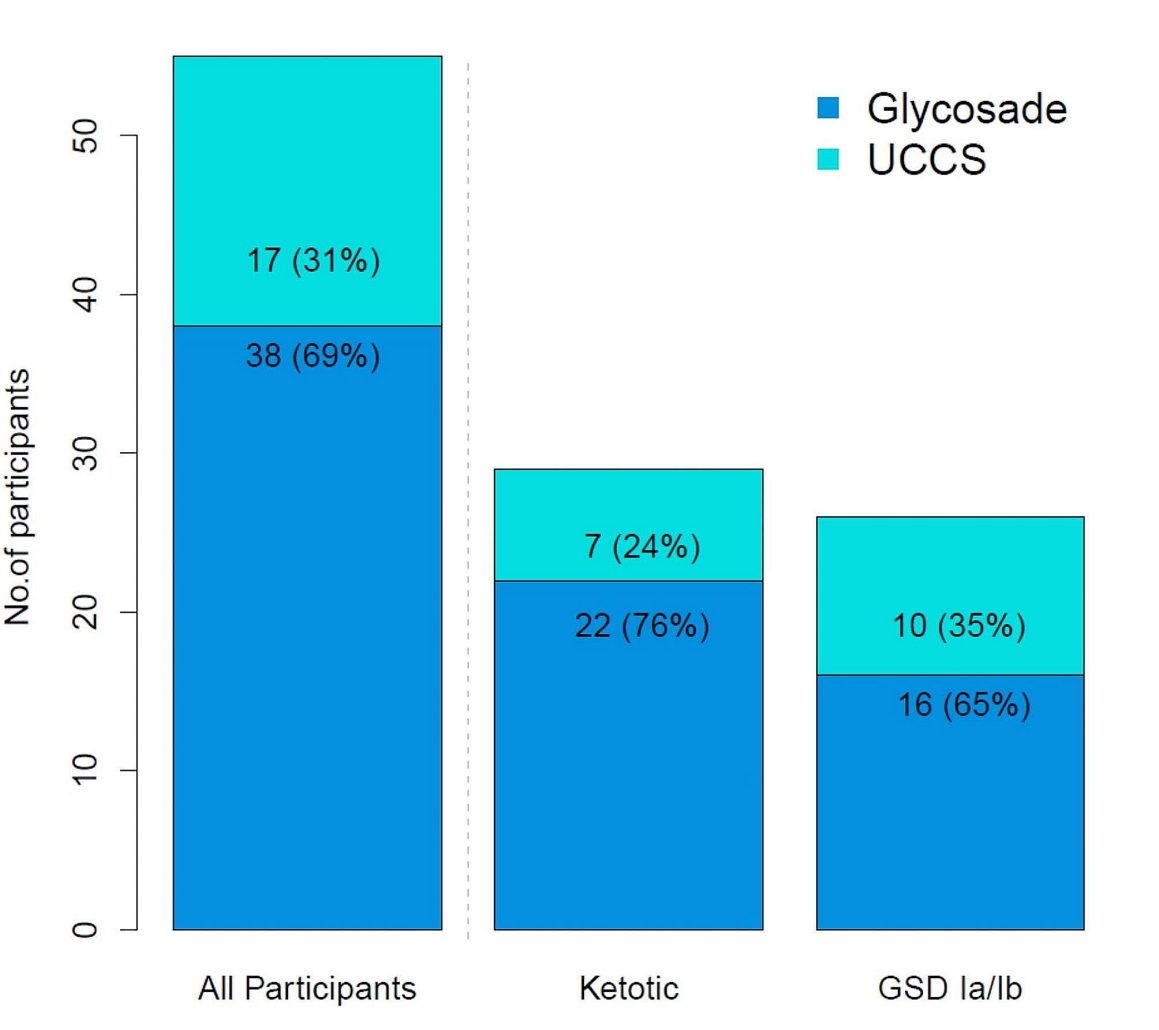
Bar plot to show the number of participants selecting Glycosade or UCCS

For all participants, the time to the peak glucose level was also greater for Glycosade (67.8 min) than for UCCS (56.4 min) although not statistically significant. The trend favouring Glycosade is consistent across age categories and ketotic GSD type. (Fig. 2 and Supplementary Table 2).

### Insulin

Profile plots of mean insulin levels over a 10-hour period for each starch product are included in Supplementary Fig. 8. The area under the curve (AUC) (95% CI) was 917 (869, 965) mmol/L for Glycosade and 895 (838, 952) mmol/L for UCCS (Table 2). Longitudinal analysis including a treatment-by-time interaction did not demonstrate any statistical significance (*p* = 0.757). There is some evidence of differential effect on ketone formation with ketotic GSD participants having a larger AUC on Glycosade [896 (823, 969)] than insulin [846 (758, 933)] although this does not reach statistical significance (*p* = 0.151, Supplementary Fig. 8).

**Table 2:** Clinical outcomes of the cross over period of the Glyde study

			Glycosade	UCCS
**All patients**	Blood Glucose	Median time ≥ 3.6 mmol/L	8.5 (6.5, > 12)	7.5 (6.0, > 12)
		Hazard Ratio (95% CI)		0.785 (0.624,0.988);
		*P*-value		**0.039**
	Insulin	Area under Curve (AUC)	917 (869, 965)	895 (838, 952)
		*P*-value		**0.757**
**GSD III/VI/IX**	Blood Glucose	Median time ≥ 3.6 mmol/L	Unobtained*	Unobtained
		Hazard Ratio (95% CI)		0.769 (0.481 : 1.229)
		*P*-value		**0.272**
	Insulin	AUC	896 (823, 969)	846 (758, 933)
		*P*-value		**0.151**
	BOHB	Median time ≤ 0.4 mmol/L	9.4 (7.4, > 12)	8.0 (7.0, 11.4)
		Hazard Ratio (95% CI)		0.671 (0.450 : 1.002)
		*P*-value		**0.05**
**GSD Ia/Ib**	Blood Glucose	Median time ≥ 3.6 mmol/L	6.5 (6.0, 7.5)	6.0 (5.0, 7.4)
		Hazard Ratio (95% CI)		0. 720 (0.533 : 0.972)
		*P*-value		**0.032**
	Insulin	AUC	940 (875, 1005)	948 (875, 1021)
		*P*-value		**0.189**

*Ketotic GSD types: blood glucose levels did not reach 3.6mmol/L

### β-hydroxybutyrate (BOHB)

The Glycosade treated group had a median time to significant ketosis of 9.4 h (7.4, > 12) which was significantly longer than the time on UCCS 8.0 h (7.0, 11.4); HR (95% CI) : 0.671 (0.450 : 1.002); *p* = 0.005,Table 2, Supplementary Fig. 7]. Further to this, it was observed 16/50 (32%) with complete BOHB profiled demonstrated delayed ketosis by at least one hour.

### Lactate

Lactate results are included in Supplementary Tables 3 and show no significant difference in the change in lactate over the cross-over period of the study between UCCS and Glycosade (*P* = 0.199).

### Choice of on-going treatment

The choice of ongoing management was a shared decision-making process involving both participant and clinician whilst blinded. This involved a review of the acceptability of the starch and biochemical parameters (blood glucose, lactate or BOHB). Overall, a significantly greater proportion of participants continued with Glycosade [38/55 (69%)] compared to UCCS [17/55 (31%); *p* = 0.006]. This was consistent across ketotic [Glycosade: 22/29 (76%) Vs UCCS: 7/29 (24%); *p* = 0.008] and GSD I participants Glycosade: 16/26 (62%) Vs UCCS: 10/26 (38%); *p* = 0.327, Fig. 2. Since published standards for dosing of Glycosade have not been published, dosing and titration of therapy for participants was performed by each team as per the standards at each institution.

### Long-term analysis

Fifty participants provided data for analysis and are categorised as either receiving Glycosade exclusively (33/50, 66%) UCCS exclusively (11/50, 22%) or some a combination of both starches (6/50, 12%). Details of starch product received at visits 4 and 5 are included in Supplementary Fig. 5. None of the 33 participants (0%) who took Glycosade exclusively around the clock opted to resume cornstarch during the 2-year long-term follow-up. In contrast, 2/12 participants (17%) who remained on cornstarch following the blinded challenges requested to commence Glycosade during the period of long-term follow-up, and 1 participant who was using both starches transitioned to Glycosade exclusively. No participant discontinued Glycosade during the long-term follow-up, and no one started cornstarch who was not on the therapy after visit 3.

Overall, there was a reduction in the number of intakes of starch required with a mean (SD) 3.95 (2.03) number of doses at baseline reducing to 3.3 (2.01) at Visit 4 and 3 (2.03) at Visit 5 (*p* = 0.013, Table 3). This trend was observed across both the GSD I (*p* = 0.109) and ketotic GSD (*p* = 0.022) populations. Fewer doses of therapy were required per day during the longitudinal part of the study for participants treated with Glycosade (Table 3a and 3b) compared with UCCS although this did not reach statistical significance. While fewer doses of therapy were administered, markers of metabolic control remained stable or improved compared with baseline (Table 4). Of note, during the course of the study, pharmacologic therapy for the participants was not modified.

**Table 3:** Summary of starch intake over the course of the study

		Baseline			Visit 4 (12 months post starch load)			Visit 5 ( 24 months post starch load)			*P* value	
		mean *±* SD	median	range	mean *±* SD	median	range	mean *±* SD	median	range		
All Part.	# of daily intakes	4 *±* 2	3	0–9	3 *±* 2	3	0–9	3 *±* 2	3	0–7		0.013
	Starch Quantity g/day	169 *±* 112	148	25–420	196 *±* 112	167.5	40–420	191 *±* 113	155	35–440		0.196
Type I	# of daily intakes	4.7 *±* 2.3	5	1–9	4.14 *±* 2.21	4	0–9	3.59 *±* 2.43	4	0–7		0.109
	Starch Quantity g/day	240 *±* 114	270	42–420	269 *±* 102	287.5	82–420	270 *±* 109	292	80–440		0.277
Type III/VI/IX	# of daily intakes	3.25 *±* 1.41	3	0–6	2.58 *±* 1.52	3	0–6	2.47 *±* 1.44	2	0–6		0.022
	Starch Quantity g/day	108 *±* 64	110	25–265	133 *±* 76	120	40–385	124 *±* 64	120	35–275		0.167
Glycosade	# of daily intakes	4 *±* 2	3	1–9	3.48 *±* 1.72	3	1–6	3.12 *±* 1.62	3	1–5		0.142
	Starch Quantity g/day	155 *±* 109	122	30–335	180 + 104	160	40–390	182 *±* 109	147.5	45–440		0.263
UCCS	# of daily intakes	4 *±* 2	3	1–9	4 *±* 2	3	2–6	3 *±* 2	3	1–7		0.947
	Starch Quantity g/day	150 *±* 117	120	30–420	203 *±* 132	158	45–420	167 *±* 115	125	35–390		0.439
UCCS -> Glycosade	# of daily intakes	3 *±* 1	3	1–6	2.87 *±* 0.99	3	1–5	2 *±* 1	3	1–5		0.671
	Starch Quantity g/day	97 *±* 75	75	25–320	123 *±* 61	120	40–270	125 *±* 64	115	55–270		0.249
UCCS -> UCCS	# of daily intakes	4 *±* 1	3	2–6	3.56 *±* 1.74	3	1–6	3 *±* 2	3	0–6		0.796
	Starch Quantity g/day	149 *±* 114	120	45–420	199 *±* 143	120	45–420	161 *±* 118	125	35–390		0.521
Both -> UCCS	# of daily intakes	5 *±* 2	5	3–9	4 *±* 2	4	2–9	4 *±* 2	4	1–9		0.050
	Starch Quantity g/day	215 *±* 103	235	40–375	242 *±* 101	250	120–390	248 *±* 101	262.5	120–440		0.399

**Table 3a:** Summary of Glycosade intakes for GSD I

	Glycosade intakes GSD Type I				
Age (years)	Day intake range g	Median # intakes per day	Median Day time intake g	Night time intake range g	Median Night time intake g
7–8	50–70	3	70	105	105
9–10	30–60	3	50	100–115	100
11–14	50–75	4	55	135–145	140
15–17	75	4	75	135–145	140
Adults (over 18yrs)	30–90	4	45	90–165	120

**Table 3b:** Summary of Glycosade intakes for ketotic GSD types

	Glycosade intakes GSD Type III, VI & IX				
Age (years)	Day intake range g	Median # intakes per day	Median Day time intake g	Night time intake range g	Median Night time intake g
2–4	15–30	2	25	-	-
5–6	20–55	2	43	-	-
7–8	20–55	2	43	-	-
9–10	20–75	2	30	60–85	65
11–14	20–75	1	20	60–90	60
15–17	45–60	1	45	60–80	60
Adults (over 18yrs)	60–80	1	60	60–80	68

**Table 4:** Summary of biochemical parameters

GSD Type	Biochemical Parameter	Units	Baseline			Visit 4			Visit 5			*P* value
			mean *±* SD	median	range	mean *±* SD	median	range	mean *±* SD	median	range	
Type Ia	ALT	U/L	43.82 *±* 36.27	27.5	11–152	37.82 *±* 31.21	24.5	13–126	32.65 *±* 23.35	24	12–98	0.299
	AST	U/L	40.13 *±* 20.25	35	21–105	43.67 *±* 37.17	28	19–178	38.95 *±* 26.26	28	15–113	0.845
	Triglycerides	mmol/L	4.31 *±* 2.7	3.48	1.44–12.7	3.87 *±* 2.21	3.02	1.06–9.46	4.32 *±* 2.78	3.42	1.34–13.4	0.72
	HDL Cholesterol	mmol/L	0.95 *±* 0.24	0.92	0.4–1.37	0.97 *±* 0.18	0.98	0.5–1.22	0.93 *±* 0.21	0.95	0.3–1.22	0.945
	LDL Cholesterol	mmol/L	2.63 *±* 1.27	2.54	0.04–4.8	3 *± +* 1.18	2.59	0.6–5.8	2.58 *±* 1.15	2.3	0.2–5.7	0.642
	Total Cholesterol	mmol/L	5.32 *±* 1.33	5.3	1.8–7.7	5.64 *±* 1.61	5.4	2.6–9.5	5.15 *±* 1.24	5.2	2.4–7.3	0.795
Type III/VI/IX	Protein	g/L	70.42 *±* 2.94	70	66–78	72.14 *±* 4.43	72	62–84	72.86 *±* 4.25	73	65–85	0.09
	ALT	U/L	151.28 *±* 175.49	88	13–687	113.07 *±* 124.33	54	14–434	132.36 *±* 150.71	71	14–673	0.428
	AST	U/L	119.71 *±* 135.14	72	21–592	84.62 *±* 86.66	56.5	18–309	111.63 *±* 119.54	82	13–493	0.453
	Triglycerides	mmol/L	1.82 *±* 0.97	1.71	0.53–5.01	1.81 *±* 1.55	1.48	0.52–8.9	2 *±* 2.02	1.59	0.52–11.2	0.791
	HDL Cholesterol	mmol/L	0.93 *±* 0.33	0.9	0.27–1.63	0.99 *± +* 0.33	1	0.39–1.89	1.05 *±* 0.3	1.01	0.31–1.76	0.208
	LDL Cholesterol	mmol/L	2.54 *±* 1.15	2.52	0.06–5.31	2.56 *±* 0.8	2.57	1.12–4.7	2.56 *±* 0.77	2.48	1.4–3.9	0.933
	Total Cholesterol	mmol/L	4.14 *±* 1.09	4.1	2.4–6.7	4.25 *±* 0.95	4.1	2.9–7	4.42 *±* 1.23	4.3	3–9.2	0.437

ALT Alanine transaminase, AST Aspartate aminotransferase

### Side effects

Side effects are partitioned to those reported in the initial treatment phase (study visits 2 & 3) and those observed during the longer phase of follow-up. During the initial starch loads, a total of 171 events were reported from 42/55 (76%) of participants. Of these, 42 participants reported 135 mild events, one participant reported 25 moderate events and one participant reported one severe event. The severe event was included as a Serious Adverse Event defined as “serotonin syndrome - non-study medications side effect” which required hospitalisation. One participant required hospitalisation, and another had a prolonged hospitalisation following visit 3 for social concerns, but modification of the therapy was not required. Of the events, 76/171 (44%) were considered to be at least ‘possibly’ related to one of the starch products.

Following Visit 3, the study was open label and, a further 47 events were reported by 23 (46%) of participants. Twenty-one participants reported 43 mild events and four participants reported 4 moderate events. Of these events, 12/47 (26%) were considered at least ‘possibly’ related to one of the starch products. Starch intakes were temporarily interrupted in 7/12 when clinical management was indicated. A single fatality was observed of “polyradiculoneuritis syndrome post infectious gastroenteritis” in a patient treated with UCCS, but the SAE was deemed by both the investigator and the independent monitor to be unrelated to the therapy. A full line listing of all SAEs observed during the long-term follow-up is included in Supplementary Table 4.

### Quality of life

Given the small number of participants, results of the ITQOL are restricted to line listings, included in Supplementary Table 5. Full summaries of all domains are included in Supplementary Tables 6 and 7. QoL scores are assessed using Analysis of Covariance and do not show any significant differences between Glycosade and UCCS on either the CHQ38 (*p* = 0.904) or SF36 (*p* = 0.112).

### Biochemistry

Details on biochemical parameters for baseline, visit 4 and visit 5 are included in Table 4 for GSD Type Ia and ketotic participants. For ketotic participants, total protein increased (although not significantly) over the course of the study with a mean (SD) level at baseline of 70.42 (2.94) g/L rising to 72.14 (4.43) g/L at Visit 4 and 72.86 (4.25) g/L Visit 5 (*p* = 0.09). There is no evidence of any difference in any other biochemical parameters showing good metabolic control.

### Anthropometry

Anthropometrical measurements are reported for height, weight, body mass index (BMI), waist/hip ratio and mean abdominal circumference. Data are stratified by patient age with analyses being conducted separately for adults and children. No significant differences were observed between study periods for the full set of participants by those choosing UCCS or Glycosade or by GSD type (Supplementary Table 8).

### Participants less than 5 years of age

Three participants were recruited into the study less than 5 years of age with ages of 24, 25 and 57 months respectively. All three participants had ketotic GSD types (one participant GSD type IIIa and two participants type GSD IX). All three participants continued with Glycosade beyond the cross-over component of the study. Details on biochemistry and starch intakes are included in Supplementary Table 9.

## Discussion

While Glycosade has been used to treat glycogen storage diseases since 2009, many questions have remained due to the limited published articles on chronic use of the therapy. Prior to initiation of this study, only 4 trials reporting on the efficacy and tolerance of Glycosade have been published [9, 12, 13]. Particular limitations of the prior studies include a lack of data on daytime use of the therapy, and minimal published experience with Glycosade in the ketotic GSD population for which only one prior article has been published [12]. While Glycosade is approved for use from 2 years of age in many countries, experience in children under 5 years of age is limited to case reports or small studies [10, 17]. It is also important to note that most of the literature has been written by the same GSD centre [12, 13], and it was deemed important to have efficacy data across a more diverse population due to regional differences in genetics and treatment. The Glyde study was created to be an international collaboration to address these limitations. In a larger and more diverse population, Glycosade was again found to be more efficacious and preferred when compared to UCCS following blinded fasting challenges. Long-term therapy was well tolerated, and metabolic control was found to be maintained with fewer doses of therapy.

Due to the severity of GSD type I, prior studies have focused on fasting tolerance due to the risk of severe hypoglycaemia associated with a missed dose when parents and patients must awaken overnight for cornstarch administration. In this study, the median fasting tolerance was again found to be statistically significantly longer in the Glycosade treated group when compared with those treated with UCCS. Of particular importance is that all participants regardless of age tolerated fasts of at least 6 h, and 82% (41/50) of participants fasted more than 8 h adding to the growing literature that use of the therapy offers the opportunity for patients to sleep the night without having to awaken for therapy. It is also important to note that these studies demonstrated better fasting tolerance with UCCS than in prior studies, and some of the patients tolerated prolonged fasting even without the extended-release formulation. This difference is likely explained by increased diversity in the patient population in this study through inclusion of a more global population. Prior studies performed in the USA had a predominance of type Ia patients with the p.R83C G6PC variant which has been found to have no enzyme activity and a more severe phenotype. The p.Q347X genetic variant which is more common in the United Kingdom is associated with partial enzyme activity (Supplementary Table 10). While these patients can often tolerate more prolonged fasts without developing symptomatic hypoglycaemia, hyperlactataemia and hyperlipidaemia can occur which can be ameliorated by optimising dietary management.

Prior published experience in the ketotic forms of GSD (GSD 0, III, VI, and IX) is limited to one publication reporting on the experience in 16 participants [12]. The Glyde study is therefore the largest study to date in this population, and clear benefits were found both during the fasting challenges and with chronic use of the therapy. The benefit in the fasting challenges is prevention of ketosis, and patients were found to have a 20% increase in the fasting tolerance prior to development of ketones. While not demonstrated in this study, prevention of ketosis may help prevent ketone induced vomiting and metabolic crises in this population. In addition, chronic ketosis may also impact growth and bone density, but the duration of this study and relatively small sample studies did not allow these to be assessed.

In addition to metabolic consequences of the therapy, it is critically important to consider the associated quality of life and the burden on patients. Following the blinded fasting challenges, a preponderance of the participants chose Glycosade for the preferred treatment in both the GSD I and ketotic GSD populations. Glycosade in GSD I again was found to allow patients to sleep through the night which is critically important for maximizing safety and improving well-being. The benefits of Glycosade during the day in the GSD I population, however, is less clearly demonstrated, and the ability to decrease the daytime support may depend on other factors including levels of physical activity, dietary intake, and baseline enzyme activity. In contrast, most patients with the ketotic forms of GSD are able to fast through the night on UCCS, but Glycosade decreased ketosis and allowed for fewer doses during the day. It is notable that 94% (16/17) of the patients who used Glycosade around the clock with the ketotic forms of GSD could use 3 or fewer doses of therapy during the day. Participants commented that this allowed avoidance of therapy at school which was deemed a particularly beneficial aspect of the therapy both medically and socially.

While this is the first international trial to collaboratively study the use of Glycosade, it is important to note that there are some limitations of this study. GSD treatment practices across the world are not standardized, and the differences in dietary therapy and consumption of non-utilizable sugars (fructose and galactose) likely added variability to the results. The goal of determining efficacy in the children under 5 years of age could not be achieved due to the small number of participants in this age group and the challenges in recruiting younger children. While the benefits in the GSD III and GSD IX population are expected to translate to the less common ketotic forms of GSD (GSD 0 and GSD VI), the efficacy in these populations could not be assessed. Similarly, there was only one participant with GSD Ib in these studies. Prior studies in GSD Ib have revealed suboptimal tolerance of Glycosade due to the underlying intestinal issues associated with this condition, but the mechanism of which is not fully understood [13]. Future studies are warranted specifically in GSD Ib since the introduction of SGLT2 inhibitors (such as empagliflozin) for treatment in GSD Ib has resulted in decreased inflammatory bowel disease and intestinal disease [18–20].

Long-term complications in all hepatic GSD types can be delayed or prevented with achievement of optimal metabolic control (Beegle, Brown, & Weinstein, [21]; Dambska, Labrador, Kuo, & Weinstein, [22]; Minarich, Kirpich, Fiske, & Weinstein, [23]; Tsilianidis et al., [24] Wang, Fiske, Carreras, & Weinstein, [25]). It is therefore imperative that outstanding metabolic control is maintained, and it is reassuring that the biochemical studies have remained stable in this cohort chronically treated with Glycosade despite administration of fewer doses of therapy. Of note, monitoring of long-term complications was not specifically included in this study since this was performed as part of clinical care, but no AEs or SAEs were reported related to liver or kidney co-morbidities. The duration of follow-up was also only 2 years, and patients treated with Glycosade should continue to be monitored closely for hepatic lesions and GSD nephropathy. It is important to also note that protein supplementation is deemed to be critically important in the ketotic forms of GSD. This is particularly true in GSD III where a higher protein intake or supplementation is deemed important for amelioration of muscle disease [26]. Thus, it will be important for patients treated with fewer doses of starch to be monitored closely for worsening of their CK concentrations and muscle symptomatology.

## Conclusions

The Glyde study is the first international trial of the efficacy and tolerance of Glycosade in a diverse international GSD population. The therapy was found consistently to not only improve the duration of tolerated fasting, but also minimise metabolic perturbations across both the ketotic and non-ketotic GSD populations. Glycosade was preferred by the majority of participants and health care professionals in a blinded fashion, and this study adds to the growing literature supporting the safety and efficacy of Glycosade for treatment of the hepatic forms of GSD.

### Electronic supplementary material

Below is the link to the electronic supplementary material.

Supplementary Material 1

Supplementary Material 2

## Data Availability

The data that support the findings of this study are available from Vitaflo (International) Ltd. upon reasonable request. The data are not publicly available due to privacy and patient confidentiality restrictions but can be obtained by contacting the Research and Development department at Vitaflo via email: clinicaltrialsteam@vitaflo.co.uk .

## Abbreviations

GSD: Glycogen Storage Disease
UCCS: Uncooked Corn starch
GSD 0: Glycogen Storage Disease Type 0
GSD I: Glycogen Storage Disease Type I
GSD Ia: Glycogen Storage Disease Type Ia
GSD Ib: Glycogen Storage Disease Type Ib
GSD III: Glycogen Storage Disease Type III
GSD IIIa: Glycogen Storage Disease Type IIIa
GSD IIIb: Glycogen Storage Disease Type IIIb
GSD VI: Glycogen Storage Disease Type VI
GSD IX: Glycogen Storage Disease Type IX
CGM: Continuous glucose monitoring
USA: United States of America
UK: United Kingdom
NRES: National Research Ethics Committee
IRAS: Integrated Research Application System
CPP: Comite de Protection des Personnes
IRB: Institutional Review Board
CCMC: Connecticut Children’s Medical Centre
METc: UMCG Universitair Medisch Ethische Toetsingscommissie: University Medical Centre Groningen
BOHB: β-hydroxybutyrate
QoL: Quality of life
ITQOL: Infant Toddler Quality of Life Questionnaire
CHQ-PF28: Child Health Questionnaire
SFv36: 36-Item Short Form Survey
AE: Adverse Event
SAE: Serious Adverse Event
BMI: Body mass index
SGLT2: Sodium-glucose co-transporter-2
ALT: Alanine transaminase
AST: Aspartate aminotransferase
TG: Triglycerides
