# Correction to: short and long-term acceptability and efficacy of extended-release cornstarch in the hepatic glycogen storage diseases: results from the Glyde study

**DOI:** 10.1186/s13023-024-03305-8

**Published:** 2024-08-30

**Authors:** DA Weinstein, RJ Jackson, EA Brennan, M Williams, JE Davison, F de Boer, TGJ Derks, C Ellerton, B Faragher, J Gribben, P Labrune, KM McKittrick, E Murphy, KM Ross, U Steuerwald, C Voillot, AJM Woodward, HR Mundy

**Affiliations:** 1 Glycogen Storage Disease Program, Connecticut Children’s Medical Center, Hartford, USA; 2 School of Medicine, Department of Pediatrics, University of Connecticut, Farmington, CT USA; 3 Liverpool Clinical Trials Centre, University of LiverpoolUK, Liverpool, UK; 4 Vitaflo International Ltd, 182 Sefton Street, Liverpool, UK; 5 Metabolic Medicine, Great Ormond Street Hospital, London, UK; 6 Division of Metabolic Diseases, Beatrix Children’s Hospital, University Medical Center Groningen, Groningen, The Netherlands; 7 Charles Dent Metabolic Unit, National Hospital for Neurology and Neurosurgery, Queen Square, London, UK; 8 Liverpool School of Tropical Medicine, Pembroke Place, Liverpool, UK; 9 Evelina London Childrens Hospital, Westminster Bridge Road, London, UK; 10 Centre de Référence des Maladies héréditaires du Métabolisme Hépatique, APHP, Hôpitaux Universitaires Paris-Saclay, Hôpital Antoine Béclère, Paris-Saclay University, Clamart, Paris, France; 11 Medical Center, National Hospital of the Faroe Islands, Tórshavn, Faroe Islands

Following publication of the original article [1], we have been notified that there was a mistake in the published articles and authors’ first and last names were published incorrectly.

They are now as follows:

Weinstein DA1,2*, Jackson RJ3, Brennan EA4, Williams M1, Davison JE5, de Boer F6, Derks TGJ6, Ellerton C7,Faragher B8, Gribben J9, Labrune P10, McKittrick KM4, Murphy E7, Ross KM1, Steuerwald U11, Voillot C10,Woodward AJM9 and Mundy HR9

They should be as follows:

DA Weinstein 1,2*, RJ Jackson 3, EA Brennan 4, M Williams1, JE Davison5, F de Boer 6, TGJ Derks 6, C Ellerton7,B Faragher 8, J Gribben 9, P Labrune 10, KM McKittrick 4, E Murphy 7, KM Ross 1, U Steuerwald 11, C Voillot 10,AJM Woodward 9 and HR Mundy 9

The original article was updated.
