# Supplementary material for: Short and long-term acceptability and efficacy of extended-release cornstarch in the hepatic glycogen storage diseases: results from the Glyde study
Source: Orphanet J Rare Dis. 2024 Jul 9;19:258. doi: 10.1186/s13023-024-03274-y (PMC11232220; doi:10.1186/s13023-024-03274-y)
Supplement: Supplementary file 1 — Supplementary Material 1 [file 13023_2024_3274_MOESM1_ESM.docx]

## Tables

**Supplementary Table 1: Withdrawal reasons / comments**

| Subject ID | GSD type | Adult or Child | Main reason for coming to the end of study | If due to an Adverse Event, please state | If due to another reason, please specify | Further comments |
| --- | --- | --- | --- | --- | --- | --- |
| 06-001 | Ia | A | Adverse Events related | painful flatulence, diarrhoea |  |  |
| 04-006 | Ia | A | Other |  | Anxiety | She is very anxious with being at hospital, and she cannot come back a second time in the same year. |
| 06-023 | IX | C | Adverse Events related | AE #1 |  | The prescribed starch from baseline was continued. Patient did not reach Visit 3. |
| 04-001 | Ia | A | Other |  | Inclusion error | Did not meet inclusion criteria |
| 05-008 | IIIb | C | Other |  | Cannulation difficulties | He did not use starch yet |
| 03-002 | Ia | A | Consent withdrawn |  |  | Approached for verbal consent to continue with patient visit. Patient & mother refused. |
| 06-015 | IX | A | Adverse Events related | Please refer to AE #1 |  | Admitted to hospital following starch load procedure as patient became lightheaded and metabolic profile was within normal range. |
| 04-003 | Ia | A | Other |  | Inclusion error | Did not meet inclusion criteria |
| 05-002 | IIIa | A | Other |  | Taste of the starch. | The patient had difficulties taking the starch, did not like the taste |

**Supplementary Table 2 – Additional Summary of blood glucose data**

|  | Median Blood Glucose  ≤3.6 UCCS (hours) | Median Blood Glucose  ≤3.6 Glycosade (hours) | % participants with an increase on Glycosade | % participants with an increase of more than 1 hour |
| --- | --- | --- | --- | --- |
| All participants | 7.5 (6, >12) | 8.5 (7.5, >12) | 53% | 42% |
| Age < 14 | 7.5 (6.6, >12) | 8.5 (7.3, >12) | 53% | 43% |
| Age > 14 | 7.2 (6.25, >12) | 7.3 (6.5, >12) | 50% | 38% |
| Ketotic | > 12 hours | > 12 hours | 55% | 50% |
| GSD Ia & Ib | 6 (5 - 7.4) | 6.5 (6- 7.5) | 55% | 33% |

**Supplementary Table 3 – Summary of Lactate data over study visits 2 and 3**

|  | Visit 2 | | | Visit 3 | | | p |
| --- | --- | --- | --- | --- | --- | --- | --- |
|  | mean + SD | median | range | mean + SD | median | range |  |
| All participants  mmol/L | 2.86 +- 1.37 | 2.77 | 0 - 5.9 | 2.4 +- 1.36 | 1.96 | 0 - 5.69 | 0.126 |
| UCCS first  mmol/L | 2.62 +- 1.24 | 2.66 | 0 - 5.3 | 2.64 +- 1.28 | 2.33 | 1.3 – 4.64 |  |
| Glycosade first  mmol/L | 3.33 +- 1.51 | 3.42 | 1.2 - 5.9 | 2.36 +- 1.34 | 2.05 | 0 - 5.69 | 0.199* |

****P value comparing UCCS Vs Glycosade using linear regression model adjusting for period and randomised group.***

**Supplementary Table 4: Line Listing of all serious adverse events**

| Subject.ID | Description | Start Date | Stop Date | Action Taken | Severity | Freq | Outcome | Relationship | starch |
| --- | --- | --- | --- | --- | --- | --- | --- | --- | --- |
| 06-012 | Participant was hospitalized for NSAID induced gastritis. While in the hospital, participant was on D10, and study product intake has been temporarily interrupted. | 29/11/2018 | 06/12/2018 | 4 - Temporarily Interrupted | 2 - Moderate | Single | 1 - Resolved | 1 - None | Glycosade |
| 06-012 | Hospitalization due to nausea, vomiting and migraine headaches | 25/03/2019 | 29/03/2019 | 4 - Temporarily Interrupted | 2 - Moderate | Single | 1 - Resolved | 1 - None | Glycosade |
| 06-010 | Planned hospital admission for a g-tube closure. | 20/02/2019 | 22/02/2019 | 4 - Temporarily Interrupted | 2 - Moderate | Single | 1 - Resolved | 1 - None | Both |
| 04-005 | hypoglycemic convulsion with loss of consciousness after a delay in taking glycosade | 16/07/2019 | 17/07/2019 | 1 – None | 3 – Severe | Single | 1 - Resolved | 1 - None | Glycosade |
| 04-005 | Hypoglycemia with convulsion during the night (1.30am) 27-28 January 2019. Capillary glycemia 1.5mmol/l | 28/01/2019 | 28/01/2019 | 1 – None | 3 – Severe | Single | 1 - Resolved | 3 - Possible | Glycosade |
| 05-001 | Potential viral infection and stress | 11/09/2018 | 13/09/2018 | 1 – None | 1 – Mild | Single | 1 - Resolved | 1 - None | Glycosade |
| 05-001 | She had complaints of fever (39-40). When she attended the GP, glucose was 3.4 mmol/L, in the ambulance 2,4 mmol/L, at the ER in the hospital 7.5 mmol/L (lactate 15.1 mmol/L). During hospitalisation the working diagnosis is gastroenteritis. | 04/07/2018 | 10/07/2018 | 4 - Temporarily Interrupted | 1 – Mild | Single | 1 - Resolved | 1 - None | Glycosade |
| 06-014 | Stroke | 20/05/2019 | 18/06/2019 | 4 - Temporarily Interrupted | 3 – Severe | Single | 2 - Resolved with sequalae | 2 - Unlikely | Glycosade |
| 01-001 | Aching Legs ? Hypoglycaemia. Symptoms resolved. Not definitely proven to be hypoglycaemia. Profile did not show hypoglycaemia. Adjustment to diet may have helped | 28/06/2017 | 02/10/2017 | 1 – None | 1 – Mild | Intermittent | 1 - Resolved | 2 - Unlikely | Glycosade |
| 02-002 | Hospital admission with diarrhoea and vomiting; this resulted in secondary hypoglycaemia and lactic acidosis. All stool cultures normal. Participant temporarily unable to take uncooked cornstarch - instead used nasogastric feed for several days and gradually weaned back onto uncooked cornstarch. | 12/03/2018 | 17/08/2018 | 4 - Temporarily Interrupted | 3 – Severe | Intermittent | 1 - Resolved | 2 - Unlikely | UCCS |
| 05-010 | Vomiting | 16/01/2020 | 18/01/2020 | 2 - Intake reduced | 1 – Mild | Single | 1 - Resolved | 2 - Unlikely | Glycosade |
| 04-008 | polyradiculoneuritis syndrome post infectious gastroenteritis | 23/08/2019 | 26/08/2019 | 1 – None | 5 - Death | Continuous | 4 - Death | 1 - None | UCCS |
| 06-003 | Suspected breast cancer | 21/05/2019 | 27/05/2019 | 1 – None | 2 - Moderate | Single | 3 - Not resolved/ongoing |  |  |
| 01-004 | 4-4 day history of increasing discomfort when gastrostomy accessed. Refusing nasogastric tube and so admitted for intravenous fluids overnight. Discharged next day but continued to complain about tube. Settled ultimately when gastrostomy tube changed back to freka from balloon device | 11/05/2018 | 09/07/2018 | 4 - Temporarily Interrupted | 1 – Mild | Single | 1 - Resolved |  |  |

**Supplementary Table 5 Line listing of participants completing Infant Toddler Quality of Life Questionnaire™ (ITQOL) for children less than 5 years (n=3)**

| Trial ID | Site | Visit | Overall Health | Physical Abilities | Growth/Development | Discomfort/Pain | Temperament/Moods | General Behaviour | Getting Along w/ Others | Gen. Health Perceptions | Parental Impact Emotional | Parental Impact - Time Scale | Global Behaviour | Change in Health | Family Cohesion |
| --- | --- | --- | --- | --- | --- | --- | --- | --- | --- | --- | --- | --- | --- | --- | --- |
| 05-004 | UMCG | 1 | 60.0 | 90.0 | 30.0 | 41.7 | 40.3 | 54.2 | 41.7 | 56.8 | 17.9 | 85.7 | 60.0 | 50.0 | 60.0 |
| 05-004 | UMCG | 4 |  | 83.3 | 15.0 | 0.0 | 56.9 | 58.3 | 50.0 | 40.9 | 0.0 | 0.0 | 100.0 | 50.0 | 100.0 |
| 05-005 | UMCG | 1 | 30.0 | 96.7 | 27.5 | 16.7 | 54.2 |  |  | 45.5 | 7.1 | 81.0 |  | 50.0 | 60.0 |
| 05-010 | UMCG | 1 | 60.0 | 90.0 | 42.5 | 58.3 | 37.5 | 54.2 | 41.7 | 50.0 | 39.3 | 76.2 | 60.0 | 50.0 | 60.0 |
| 05-010 | UMCG | 4 | 60.0 | 96.7 | 40.0 | 8.3 | 43.1 | 56.3 | 45.0 | 40.9 | 25.0 | 66.7 | 60.0 | 50.0 | 60.0 |
| 05-010 | UMCG | 5 | 30.0 | 93.3 | 37.5 | 50.0 | 34.7 |  |  | 38.6 | 25.0 | 81.0 |  |  | 60.0 |

**Supplementary Table 6 Summary of QoL outcomes for 36-Item Short Form Survey (SFv36 for adults (n=34).**

| Outcome | Time | UCCS (n=3) | Glycosade (n=9) | Both (n=2) | Total |
| --- | --- | --- | --- | --- | --- |
| Physical Functioning | Baseline | 65 (57.5, 75) | 80 (35, 100) | 50 (27.5, 72.5) | 80 (50, 95) |
|  | Visit 4 | 92.5 (88.8, 96.2) | 92.5 (71.2, 95) | 50 (27.5, 72.5) | 92.5 (71.2, 95) |
|  | Visit 5 | 92.5 (88.8, 96.2) | 90 (60, 100) | 50 (27.5, 72.5) | 90 (60, 100) |
| Role Limitations (Physical) | Baseline | 68.8 (56.2, 84.4) | 62.5 (56.2, 93.8) | 68.8 (53.1, 84.4) | 62.5 (56.2, 100) |
|  | Visit 4 | 75 (62.5, 87.5) | 78.1 (48.4, 89.1) | 62.5 (43.8, 81.2) | 78.1 (48.4, 95.3) |
|  | Visit 5 | 75 (62.5, 87.5) | 87.5 (56.2, 100) | 62.5 (43.8, 81.2) | 87.5 (50, 100) |
| Role Limitations (Emotional) | Baseline | 100 (75, 100) | 91.7 (58.3, 100) | 100 (100, 100) | 100 (58.3, 100) |
|  | Visit 4 | 100 (100, 100) | 95.8 (77.1, 100) | 87.5 (85.4, 89.6) | 95.8 (83.3, 100) |
|  | Visit 5 | 100 (100, 100) | 75 (58.3, 100) | 87.5 (85.4, 89.6) | 91.7 (66.7, 100) |
| Energy/Vitality | Baseline | 70 (52.5, 75) | 55 (55, 70) | 57.5 (53.8, 61.2) | 55 (50, 70) |
|  | Visit 4 | 75 (67.5, 82.5) | 52.5 (33.8, 62.5) | 62.5 (56.2, 68.8) | 57.5 (46.2, 71.2) |
|  | Visit 5 | 75 (67.5, 82.5) | 55 (35, 70) | 55 (55, 55) | 55 (50, 70) |
| Wellbeing | Baseline | 84 (62, 86) | 66 (57, 74) | 76 (76, 76) | 70 (57, 80) |
|  | Visit 4 | 84 (82, 86) | 64 (54, 73) | 64 (62, 66) | 66 (60, 77) |
|  | Visit 5 | 86 (85, 87) | 72 (44, 76) | 60 (56, 64) | 72 (52, 80) |
| Social Functioning | Baseline | 87.5 (62.5, 93.8) | 75 (75, 100) | 75 (62.5, 87.5) | 87.5 (75, 100) |
|  | Visit 4 | 81.2 (71.9, 90.6) | 75 (62.5, 75) | 75 (62.5, 87.5) | 75 (62.5, 81.2) |
|  | Visit 5 | 81.2 (71.9, 90.6) | 75 (62.5, 100) | 56.2 (34.4, 78.1) | 75 (62.5, 100) |
| Bodily Pain | Baseline | 57.5 (51.2, 78.8) | 77.5 (67.5, 90) | 95 (92.5, 97.5) | 90 (67.5, 100) |
|  | Visit 4 | 78.8 (68.1, 89.4) | 77.5 (66.9, 100) | 95 (92.5, 97.5) | 88.8 (66.9, 100) |
|  | Visit 5 | 78.8 (68.1, 89.4) | 77.5 (67.5, 80) | 95 (92.5, 97.5) | 77.5 (67.5, 90) |
| Global Health | Baseline | 35 (32.5, 42.5) | 60 (46.2, 72.5) | 57.5 (53.8, 61.2) | 55 (42.5, 67.5) |
|  | Visit 4 | 62.5 (51.2, 73.8) | 50 (30, 76.2) | 35 (32.5, 37.5) | 40 (30, 76.2) |
|  | Visit 5 | 67.5 (58.8, 76.2) | 65 (40, 75) | 52.5 (43.8, 61.2) | 65 (40, 75) |

**Supplementary Table 7 Summary of QoL outcomes for Child Health Questionnaire™ (CHQ-PF28) for children aged 6 – 18 years (n=14)**

| Outcome | Time | UCCS (n=8) | Glycosade (n=22) | Both (n=4) | Total (n=34) |
| --- | --- | --- | --- | --- | --- |
| Global Health | Baseline | 85 (72.5, 92.5) | 85 (60, 85) | 92.5 (78.8, 100) | 85 (60, 85) |
|  | Visit 4 | 85 (85, 85) | 85 (85, 100) | 92.5 (78.8, 100) | 85 (85, 100) |
|  | Visit 5 | 85 (85, 92.5) | 85 (85, 100) | 72.5 (60, 88.8) | 85 (85, 100) |
| Physical Functioning | Baseline | 77.8 (27.8, 100) | 88.9 (77.8, 100) | 83.3 (69.4, 91.7) | 88.9 (77.8, 100) |
|  | Visit 4 | 88.9 (83.3, 100) | 88.9 (55.6, 100) | 94.4 (88.9, 100) | 88.9 (66.7, 100) |
|  | Visit 5 | 77.8 (47.2, 100) | 77.8 (55.6, 100) | 94.4 (88.9, 100) | 88.9 (55.6, 100) |
| RS - Emot./Behav. Difficulties | Baseline | 100 (83.3, 100) | 100 (100, 100) | 100 (100, 100) | 100 (100, 100) |
|  | Visit 4 | 100 (100, 100) | 100 (66.7, 100) | 100 (100, 100) | 100 (100, 100) |
|  | Visit 5 | 100 (100, 100) | 100 (100, 100) | 100 (100, 100) | 100 (100, 100) |
| RS - Physical Health | Baseline | 100 (66.7, 100) | 100 (100, 100) | 100 (100, 100) | 100 (100, 100) |
|  | Visit 4 | 100 (100, 100) | 100 (66.7, 100) | 100 (100, 100) | 100 (66.7, 100) |
|  | Visit 5 | 100 (100, 100) | 100 (83.3, 100) | 100 (100, 100) | 100 (100, 100) |
| Bodily Pain | Baseline | 80 (60, 80) | 80 (60, 100) | 80 (70, 85) | 80 (60, 100) |
|  | Visit 4 | 80 (70, 90) | 80 (80, 100) | 80 (80, 85) | 80 (80, 100) |
|  | Visit 5 | 60 (60, 70) | 80 (80, 90) | 90 (80, 100) | 80 (60, 95) |
| Behaviour | Baseline | 65 (55.6, 77.5) | 83.7 (71.2, 87.5) | 61.9 (57.2, 71.2) | 76.2 (63.4, 87.5) |
|  | Visit 4 | 74.4 (66.6, 82.2) | 81.2 (58.8, 90) | 58.8 (50.9, 72.2) | 74.4 (58.8, 88.1) |
|  | Visit 5 | 71.2 (54.1, 83.7) | 80.6 (75.6, 86.6) | 76.2 (63.4, 87.5) | 80.6 (63.4, 87.5) |
| Global Behaviour | Baseline | 85 (60, 85) | 60 (60, 85) | 85 (78.8, 85) | 85 (60, 85) |
|  | Visit 4 | 85 (72.5, 85) | 60 (0, 85) | 72.5 (45, 85) | 60 (0, 85) |
|  | Visit 5 | 60 (60, 85) | 60 (15, 85) | 42.5 (0, 85) | 60 (7.5, 85) |
| Mental Health | Baseline | 75 (66.7, 95.8) | 83.3 (66.7, 100) | 66.7 (56.2, 79.2) | 83.3 (66.7, 100) |
|  | Visit 4 | 75 (62.5, 100) | 91.7 (75, 91.7) | 79.2 (68.8, 83.3) | 83.3 (75, 91.7) |
|  | Visit 5 | 75 (70.8, 91.7) | 83.3 (66.7, 100) | 83.3 (72.9, 93.8) | 79.2 (66.7, 100) |
| Self Esteem | Baseline | 91.7 (79.2, 100) | 83.3 (75, 100) | 87.5 (75, 91.7) | 91.7 (75, 100) |
|  | Visit 4 | 95.8 (91.7, 100) | 91.7 (75, 100) | 75 (70.8, 77.1) | 91.7 (75, 100) |
|  | Visit 5 | 79.2 (75, 95.8) | 83.3 (62.5, 95.8) | 83.3 (81.2, 87.5) | 83.3 (75, 100) |
| Gen. Health Perceptions | Baseline | 31.2 (31.2, 34.4) | 37.5 (25, 50) | 31.2 (18.8, 45.3) | 37.5 (25, 45.3) |
|  | Visit 4 | 31.2 (25, 43.8) | 37.5 (31.2, 53.1) | 40.6 (26.6, 50) | 37.5 (25, 50) |
|  | Visit 5 | 37.5 (31.2, 46.9) | 37.5 (25, 46.9) | 40.6 (37.5, 50) | 37.5 (31.2, 48.4) |
| Change in Health | Baseline | 75 (62.5, 75) | 50 (50, 75) | 75 (68.8, 75) | 75 (50, 75) |
|  | Visit 4 | 75 (62.5, 75) | 50 (50, 75) | 75 (68.8, 81.2) | 75 (50, 75) |
|  | Visit 5 | 50 (50, 87.5) | 50 (50, 75) | 62.5 (43.8, 81.2) | 50 (50, 75) |
| Emotional Impact on Parent | Baseline | 75 (50, 81.2) | 75 (50, 75) | 81.2 (62.5, 90.6) | 75 (50, 87.5) |
|  | Visit 4 | 68.8 (50, 96.9) | 75 (50, 93.8) | 68.8 (43.8, 87.5) | 75 (50, 87.5) |
|  | Visit 5 | 75 (53.1, 96.9) | 62.5 (37.5, 81.2) | 100 (87.5, 100) | 68.8 (46.9, 100) |
| Time Impact on Parent | Baseline | 25 (18.8, 62.5) | 62.5 (50, 75) | 68.8 (59.4, 75) | 62.5 (50, 75) |
|  | Visit 4 | 75 (46.9, 75) | 62.5 (50, 75) | 68.8 (59.4, 75) | 68.8 (50, 75) |
|  | Visit 5 | 68.8 (53.1, 75) | 75 (50, 75) | 75 (62.5, 75) | 75 (50, 75) |
| Family Activities | Baseline | 50 (50, 87.5) | 75 (50, 100) | 87.5 (71.9, 100) | 75 (50, 100) |
|  | Visit 4 | 75 (56.2, 93.8) | 81.2 (59.4, 100) | 100 (90.6, 100) | 87.5 (62.5, 100) |
|  | Visit 5 | 81.2 (75, 96.9) | 75 (53.1, 100) | 100 (87.5, 100) | 75 (68.8, 100) |
| Family Cohesion | Baseline | 85 (60, 85) | 85 (60, 85) | 85 (85, 88.8) | 85 (60, 85) |
|  | Visit 4 | 85 (66.2, 85) | 85 (78.8, 100) | 92.5 (78.8, 100) | 85 (72.5, 100) |
|  | Visit 5 | 85 (85, 96.2) | 85 (60, 100) | 85 (85, 92.5) | 85 (78.8, 100) |

**Supplementary Table 8. Summary of the change in anthropometry parameters over the course of the study for adults and children**

|  |  | Baseline | | | Visit 4 | | | Visit 5 | | |
| --- | --- | --- | --- | --- | --- | --- | --- | --- | --- | --- |
|  |  | Mean (Sd) | Med. | Range | Mean (Sd) | Med. | Range | Mean (Sd) | Med. | Range |
| Children | Weight (kg) | 40.74 +- 19.63 | 36.9 | (11.7, 99.9) | 45 +- 20.89 | 40.2 | (13.6, 102.4) | 46.93 +- 20.94 | 44.5 | (0.1, 94.4) |
|  | Height (m) | 1.36 +- 0.23 | 1.36 | (0.82, 1.85) | 1.41 +- 0.22 | 1.43 | (0.92, 1.83) | 1.42 +- 0.29 | 1.47 | (0.1, 1.83) |
|  | BMI | 20.72 +- 4.31 | 20.4 | (14.8, 32.6) | 21.25 +- 4.68 | 20.4 | (14.4, 32.3) | 21.27 +- 5.02 | 21.1 | (10, 32.5) |
|  | Weight (Centile) | 69.27 +- 29.45 | 84 | (7, 100) | 63.62 +- 33.51 | 75 | (2, 99) | 62.83 +- 33.35 | 68.5 | (0, 99) |
|  | Height (Centile) | 47.86 +- 30.67 | 43 | (2, 98) | 46.47 +- 31.39 | 43 | (0.1, 98) | 48.86 +- 30.14 | 47 | (0, 99) |
|  | MAC (cm) | 71.11 +- 14.05 | 68.7 | (47.5, 107.7) | 73.59 +- 13.96 | 72.6 | (51.4, 108.6) | 75.44 +- 14.37 | 73.1 | (53.9, 106.5) |
|  | Waist (cm) | 65.17 +- 27.84 | 65.9 | (0, 92.2) | 87.02 +- 16.69 | 87.8 | (58, 109.5) | 84.44 +- 16.39 | 92 | (62, 102.7) |
|  | Hip (cm) | 70.09 +- 31.25 | 76.5 | (0, 102.3) | 100.86 +- 13.54 | 97.3 | (89, 123.4) | 88.5 +- 19.95 | 98.9 | (57, 105) |
| Adults | Weight (kg) | 71.69 +- 15.51 | 65.3 | (58.3, 104.6) | 75.75 +- 17.08 | 72.5 | (55.7, 111.1) | 73.73 +- 16.4 | 70.3 | (56.2, 109.1) |
|  | Height (m) | 1.63 +- 0.09 | 1.64 | (1.49, 1.8) | 1.62 +- 0.09 | 1.63 | (1.52, 1.81) | 1.62 +- 0.09 | 1.63 | (1.52, 1.81) |
|  | BMI | 27.19 +- 5.9 | 25.3 | (22, 43) | 28.66 +- 6.26 | 27.7 | (23.3, 45.1) | 27.9 +- 6.03 | 26.3 | (23.5, 44.3) |
|  | MAC (cm) | 95.98 +- 9.5 | 95.4 | (83, 117.9) | 98.91 +- 11.4 | 96.2 | (89.6, 128.2) | 94.88 +- 6.77 | 93.3 | (84, 104) |
|  | Waist (cm) | 94.7 +- 8.93 | 93 | (81.7, 113.8) | 96.47 +- 7.66 | 94.4 | (88.9, 115.4) | 95.19 +- 4.57 | 96 | (87.5, 101.5) |
|  | Hip (cm) | 101.91 +- 10.62 | 101 | (88, 124.5) | 103.69 +- 10.51 | 103 | (87, 127) | 99.9 +- 9.25 | 101 | (83.5, 111) |

**Supplementary Table 9 Line Listing of biochemistry and starch delivery for participants under 5 years of age**

| Subject ID | Age (Months) | GSD type | Visit | Protein  g/L | ALT  U/L | AST  U/L | TG  mmol/L | Total Chol.  mmol/L | HDL  Chol.  mmol/L | LDL  Chol.  mmol/L | No. of starch intakes/day | Mean starch intake  g/day |
| --- | --- | --- | --- | --- | --- | --- | --- | --- | --- | --- | --- | --- |
| 01-005 | 57 | IIIa | 1 | 70 | 632 | 592 | 3.62 | 5.3 | 0.27 | 3.4 | 2 | 40 |
|  |  |  | 4 | 69 | 434 | 309 | 2.97 | 4.9 | 0.39 | 2.97 | 2 | 40 |
|  |  |  | 5 | 74 | 673 | 493 | 2.81 | 4.4 | 0.31 | 3.83 | 2 | 45 |
| 05-004 | 25 | IX | 1 | 72 | 113 | 84 | 2.26 | 6.1 | 0.6 | 4.8 | 2 | 35 |
|  |  |  | 4 | 73 | 83 | 61 | 2.23 | 5.8 | 0.7 | 4.7 | 2 | 40 |
|  |  |  | 5 | withdrawn | | | | | | | | |
| 05-010 | 24 | IX | 1 | 72 | 108 | 97 | 3.35 | 3.6 | 0.5 | 2.3 | 3 | 46 |
|  |  |  | 4 | 71 | 24 | 33 | 0.72 | 3.1 | 1 | 2 | 3 | 60 |
|  |  |  | 5 | 69 | 20 | 30 | 1.01 | 3 | 1 | 1.8 | 3 | 85 |

ALT Alanine transaminase, AST Aspartate aminotransferase, TG Triglycerides

**Supplementary Table 10 Genetic Mutation Data**

| Subject ID | Gender | Type of GSD | Gene | Variant(s) |
| --- | --- | --- | --- | --- |
| 01-007 | Female | Ia | G6PC | homozygous c.150_151del p.(Trp50Cysfs*10) |
| 01-011 | Male | Ia | G6PC | ? Compound heterozygote c.231-2A>G , c.969C>A |
| 01-001 | Female | Ia | G6PC | Compound heterozygous c.247C>T p.(Arg83Cys) & c.1039C>T p.(Gln347*) |
| 03-001 | Male | Ia | G6PC | homozygous c.150_151del p.(Trp50Cysfs*10) |
| 03-002 | Female | Ia | G6PC | homozygous c.150_151del p.(Trp50Cysfs*10) |
| 03-003 | Female | Ia | G6PC | homozygous c.150_151del p.(Trp50Cysfs*10) |
| 04-002 | Male | Ia | G6PC | Homozygous (c.1039C>T) [p.Gln347*] |
| 04-005 | Male | Ia | G6PC | Compound heterozygote (c.562G>C) splicing & (c.1012G>T) [p.Val338Phe] |
| 04-007 | Male | Ia | G6PC | Compound heterozygous (c.208T>C) [p.Trp70Arg] & (c.1072T>C) [p.*358Gln] |
| 04-008 | Female | Ia | G6PC | Homozygous (c.247C>T) [p.Arg83Cys] |
| 05-001 | Female | Ia | G6PC | homozygous c.79delC p.(Gln27Argfs*9) |
| 05-009 | Female | Ia | G6PC | Compound heterozygote c.79delC p.(Gln27Argfs*9) & c.209G>A p.(Trp70*) |
| 06-001 | Male | Ia | G6PC | Compound heterozygote c.247C>T p.(Arg83Cys) & c.1039C>T p.(Gln347*) |
| 06-005 | Male | Ia | G6PC | Compound heterozygote c.247C>T p.(Arg83Cys) & c.883C>T p.(Arg295Cys) |
| 06-007 | Male | Ia | G6PC | compound heterozygote c.247C>T p.(Arg83Cys) & c.230+1G>C |
| 06-008 | Female | Ia | G6PC | Homozygous c.247C>T p.(Arg83Cys) |
| 06-009 | Female | Ia | G6PC | Compound heterozygote c.79delC p.(Gln27*fs?) & c.1039C>T p.(Gln347*) |
| 06-010 | Female | Ia | G6PC | Compound heterozygote c.247C>T p.(Arg83Cys) & c.562G>C p.(Gly188Arg) |
| 06-013 | Male | Ia | G6PC | Compound heterozygote c.247C>T p.(Arg83Cys) & c.328G>A p.(Glu110Lys) |
| 06-016 | Female | Ia | G6PC | Compound heterozygote c.247C>T p.(Arg83Cys) & other variant (not identified) |
| Subject ID | **Gender** | **Type of GSD** | **Gene** | **Variant(s)** |
| 06-021 | Female | Ia | G6PC | Compound heterozygote c.562G>C p.(Gly188Arg) & c.1039C>T, p.(Gln347*) |
| 06-022 | Female | Ia | G6PC | homozygous c.1039C>T p.(Gln347*) |
| 06-024 | Male | Ia | G6PC | homozygous c.648G>T p.(Leu216=) |
| 03-004 | Male | Ia | G6PC (G6PT) | homozygous c.150_151del p.(Trp50Cysfs*10) |
| 02-002 | Female | Ib | SLC37A4 | Homozygous c.1123+3_+6delAAGT |
| 01-005 | Female | IIIa | AGL | compound heterozygote c.2144delA p.(Lys715Argfs*15) & c.3682C>T p.(Arg1228*) |
| 01-003 | Male | IIIa | AGL | compound heterozygous c.3014delG p.(Cys1005Phefs*7) & c.4529dupA p.(Tyr1510*) |
| 01-010 | Male | IIIa | AGL | Homozygous c.3911dupA p.(Asn1304Lysfs*7) |
| 01-006 | Male | IIIa | AGL | homozygous c.4260-12A>G |
| 01-008 | Male | IIIa | AGL | homozygous c.4260-12A>G |
| 05-002 | Male | IIIa | AGL | ? Homozygous c.3911delA p.(Asn1304Ilefs*10) |
| 05-003 | Male | IIIa | AGL | Homozygous c.3911delA p.(Asn1304Ilefs*10) |
| 05-007 | Female | IIIa | AGL | homozygous c.1020delA p.(Glu340Aspfs*9) |
| 06-002 | Female | IIIa | AGL | Compound heterozygous c.4221dupA p.(Leu1408Ilefs*14) & c.4260-12A>G |
| 06-006 | Female | IIIa | AGL | compound heterozygote c.276delG p.(Gln92Hisfs*16) & other variant (not identified) |
| 06-014 | Female | IIIa | AGL | Not available |
| 06-018 | Female | IIIa | AGL | Homozygous, c.3965del, p.Val1322Alafs*27 |
| 06-025 | Male | IIIa | AGL | Homozygous c.3911dupA p.(Asn1304Lysfs*7) |
| Subject ID | **Gender** | **Type of GSD** | **Gene** | **Variant(s)** |
| 06-003 | Female | IIIa | AGL | compound heterozygous c.3980G>A p.(Trp1327*) & c.4529dupA p.Tyr1510* |
| 01-009 | Male | VI | PYGL | compound heterozygote c.1729C>T p.(Gln577*) and c.2416A>T p.(Ile806Leu) |
| 01-004 | Male | IX | PHKA2 | Hemizygous c.963C>A p.(Phe321Leu) |
| 01-002 | Male | IX | PHKA2 | hemizygous c.884G>A p.(Arg295His) |
| 05-004 | Male | IX | PHKA2 | hemizygous c.3614C>T p.(Pro1205Leu) |
| 05-005 | Male | IX | PHKA2 | hemizygous c.3614C>T p.(Pro1205Leu) |
| 05-006 | Male | IX | PHKA2 | hemizygous c.601T>C p.(Ser201Pro) |
| 06-004 | Male | IX | PHKA2 | Hemizygous c.133C>T p.(Arg45Trp) |
| 06-017 | Male | IX | PHKA2 | hemizygous c.4C>G p.(Arg2Gly) |
| 06-020 | Male | IX | PHKA2 | hemizygous c.750_752delGAC (p.Thr251del) |
| 06-026 | Male | IX | PHKA2 | Hemizygous c.1562insA, insertion of one A at the start of codon 521 AACA>ACA |
| 06-011 | Male | IX | PHKA2 PHKB | PHKA2: hemizygous c.893G>C p.(Arg298Pro) PHKB: carrier c.555G>T p.(Met185Ile) |
| 06-012 | Female | IX | PHKG2 | compound heterozygous c.899G>A p.(Trp300*) & c890A>G p.Tyr358Cys |
| 05-010 | Male | IX | Unknown | Diagnosed clinically and enzymatically |
| 05-011 | Male | IX | Unknown | Diagnosed clinically and enzymatically |

## Figures

**Supplementary Figure 1: Flow chart of study design**

**Supplementary Figure 2 Study Design**

**Supplementary figure 3 – Standardised Starch Load Procedures**

Perform a standard starch load as below:

1. Insert cannula and ensure venous access.
2. Unblinded personnel to weigh out and mix the test starch. This must be prepared in a separate room to protect the blinding of the study. This will minimise the identification of the test starch by either the patient or clinician.
3. Give breakfast - ensure the **exact same breakfast in terms of carbohydrate and protein quantity** (grams) is given at each loading test.
4. Commence load between 07:30am and 9:00am (note all starch loads for an individual patient must start within 30 minutes of each other).
5. **INTAKE:** Prescribe 2g carbohydrate/kg (ideal body weight for height –of test starch of either UCCS (standard, supplied by Vitaflo) or Glycosade®. **Each intake must be consumed by the participant within 15 minutes of it being administered.**
6. An acceptability questionnaire will be given to the patient and should be completed immediately after consumption of the test starch to assess taste and tolerance.
7. It is important that during each starch loading test the patient must maintain the same level of activity.
8. Measure (as per local clinical practice) at 30-minute intervals for the first 2 hours and hourly or more frequently if clinically indicated thereafter until the end of 12 hours:
   1. Plasma glucose, insulin and beta hydroxybutyrate (BOHB) (for patients with GSD III, VI and IX), *or*
   2. Plasma glucose, insulin and lactate (for patients with GSD I).
9. Continue study until either:
10. 12 hours (if no hypoglycaemia) have elapsed, *or*
11. hypoglycaemia as defined by plasma glucose <3.6mmol/L has occurred, *or*
12. the patient / parent / carer requests end of load (reason will be recorded in the patient’s record)

Once the data has been collected the patient is to return to their pre-study dietary management.

**Supplementary Figure 4: Profile plot of blood glucose participants with GSD I whilst receiving a) Glycosade and b) UCCS.**

***Figure 4a: each line represents an individual participant***

***Figure 4b***

**Supplementary Figure 5: Profile plot of individual participants blood glucose levels with GSD III, VI & IX whilst receiving a) Glycosade and b) UCCS.**

***Figure 5a – each line represents an individual participant***

***Figure 5b***

**Supplementary Figure 6: Kaplan Meier plot to show the time to failure of normal blood glucose control for a) ketotic participants and b) GSD I participants.**

**Figure 6a GSD III/VI/IX**


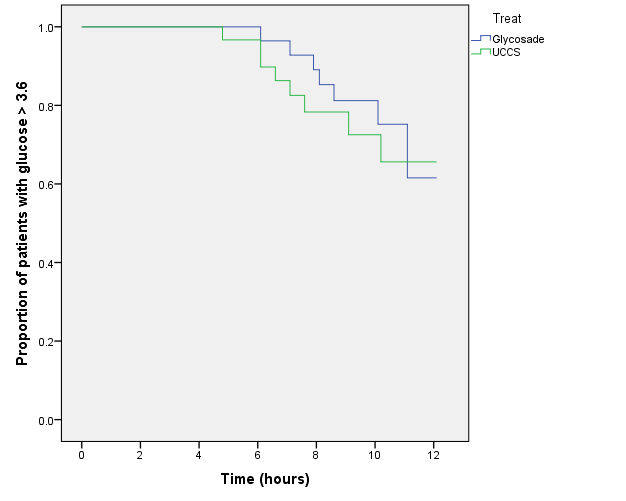


**Figure 6b GSD I**


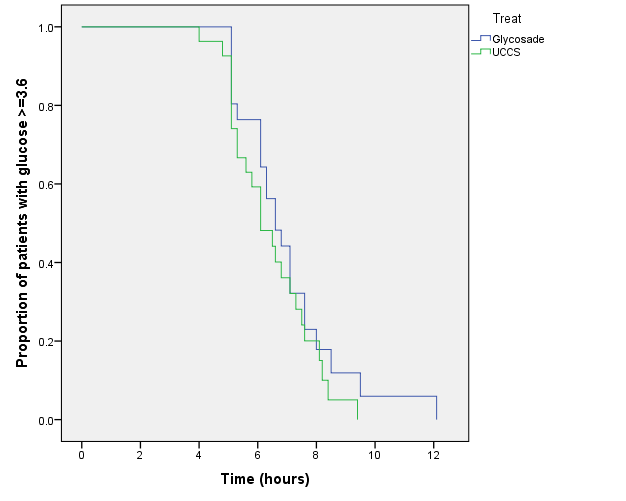


**Supplementary figure 7 Time to the development of severe ketosis (BHOB ≥0.4 mmol/l) for all ketotic patients.**


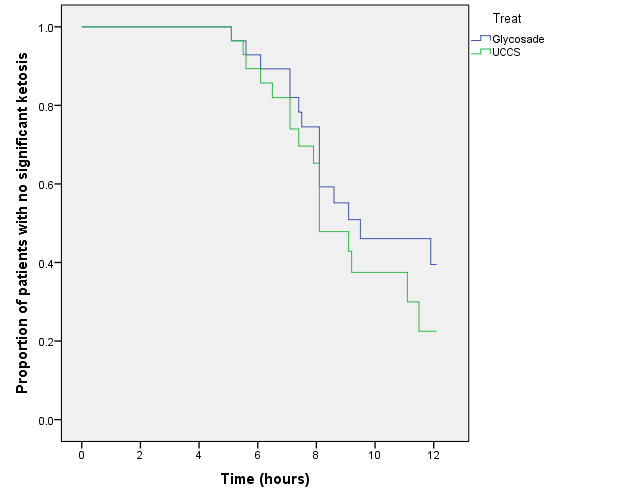


**Supplementary Figure 8: Profile plot: mean insulin levels whilst receiving Glycosade and UCCS for a) ketotic and b) GSD I participants.**

**Figure 8a**

Figure 8b

**Supplementary Figure 9: Distribution of patients across starch products for long-term analysis**

U

C

C

S

(

n

=

1

2

)

G

l

y

c

o

s

a

d

e

(

n

=

3

3

)

B

o

t

h

(

n

=

4

)

U

C

C

S

(

n

=

1

0

)

G

l

y

c

o

s

a

d

e

(

n

=

3

4

)

B

o

t

h

(

n

=

3

)

U

C

C

S

(

n

=

1

0

)

G

l

y

c

o

s

a

d

e

(

n

=

3

2

)

B

o

t

h

(

n

=

6

)

**V**

**I**

**S**

**I**

**T**

**5**

**V**

**I**

**S**

**I**

**T**

**4**

**T**

**O**

**T**

**A**

**L**

n

=

9

n

=

1

n

=

1

n

=

1

n

=

3

2

n

=

3

n

=

1

n

=

1

VISIT 4
