# Supplementary material for: Short and long-term acceptability and efficacy of extended-release cornstarch in the hepatic glycogen storage diseases: results from the Glyde study
Source: Orphanet J Rare Dis. 2024 Jul 9;19:258. doi: 10.1186/s13023-024-03274-y (PMC11232220; doi:10.1186/s13023-024-03274-y)
Supplement: Supplementary file 2 — Supplementary Material 2 [file 13023_2024_3274_MOESM2_ESM.docx]

# Tables and Figures

## Tables

**Table 1: Patient Demographics**

|  | | **Evaluable patients only** |
| --- | --- | --- |
| ***All patients*** | |  |
| Date of study entry | | 23/02/16 : 25/06/18 |
| Age (years) | median [range] | 12.0 [ 2.0 : 62.8] |
| Age Category: – Child (<14 years) | n (%) | 24 (41) |
| - Adult (>14 years) | n (%) | 34 (59) |
| Sex – Female | n (%) | 24 (41) |
| Male | n (%) | 34 (59) |
| Type of GSD: Ia | n (%) | 27(46) |
| Ib | n (%) | 1 (2) |
| IIIa / IIIb | n (%) | 15 (26) |
| IX | n (%) | 14 (24) |
| VI | n (%) | 1 (2) |
| Baseline starch: UCCS | n (%) | 34 (59) |
| Glycosade | n (%) | 6 (10) |
| Combination of both | n (%) | 18 (31) |
| Baseline Medications: GSD I |  |  |
| Uric Acid lowering | n (%) | 12 (44) |
| Kidney Stone Prophylaxis | n (%) | 4 (15) |
| Lipid lowering medications | n (%) | 2 (7%) |
| Hypertension medications | n (%) | 6 (22) |

**Table 2: Clinical Outcomes of the cross over period of the Glyde study**

|  |  |  | **Glycosade** | **UCCS** |
| --- | --- | --- | --- | --- |
| **All patients** | Blood Glucose | Median time ≥3.6 mmol/L | 8.5 (6.5, >12) | 7.5 (6.0, >12) |
|  |  | Hazard Ratio (95% CI) |  | 0.785 (0.624,0.988); |
|  |  | P-value |  | **0.039** |
|  | Insulin | Area under Curve (AUC) | 917 (869, 965) | 895 (838, 952) |
|  |  | P-value |  | **0.757** |
| **GSD III/VI/IX** | Blood Glucose | Median time ≥3.6 mmol/L | Unobtained* | Unobtained |
|  |  | Hazard Ratio (95% CI) |  | 0.769 (0.481 : 1.229) |
|  |  | P-value |  | **0.272** |
|  | Insulin | AUC | 896 (823, 969) | 846 (758, 933) |
|  |  | P-value |  | **0.151** |
|  | BOHB | Median time ≤ 0.4 mmol/L | 9.4 (7.4, >12) | 8.0 (7.0, 11.4) |
|  |  | Hazard Ratio (95% CI) |  | 0.671 (0.450 : 1.002) |
|  |  | P-value |  | **0.05** |
| **GSD Ia/Ib** | Blood Glucose | Median time ≥3.6 mmol/L | 6.5 (6.0, 7.5) | 6.0 (5.0, 7.4) |
|  |  | Hazard Ratio (95% CI) |  | 0. 720 (0.533 : 0.972) |
|  |  | P-value |  | **0.032** |
|  | Insulin | AUC | 940 (875, 1005) | 948 (875, 1021) |
|  |  | P-value |  | **0.189** |

**Ketotic GSD types: blood glucose levels did not reach 3.6mmol/L*

**Table 3: Summary of starch intake over the course of the study.**

|  |  | Baseline | | | Visit 4 (12 months post starch load) | | | Visit 5 ( 24 months post starch load) | | | P value | |  |
| --- | --- | --- | --- | --- | --- | --- | --- | --- | --- | --- | --- | --- | --- |
|  |  | mean +SD | median | range | mean +SD | median | range | mean +SD | median | range | |  | |
| All Part. | # of daily intakes | 4 + 2 | 3 | 0 - 9 | 3 + 2 | 3 | 0 - 9 | 3 + 2 | 3 | 0 - 7 | | 0.013 | |
|  | Starch Quantity g/day | 169 + 112 | 148 | 25 - 420 | 196 + 112 | 167.5 | 40 - 420 | 191 +113 | 155 | 35 - 440 | | 0.196 | |
| Type I | # of daily intakes | 4.7 + 2.3 | 5 | 1 - 9 | 4.14 +2.21 | 4 | 0 - 9 | 3.59 + 2.43 | 4 | 0 - 7 | | 0.109 | |
|  | Starch Quantity  g/day | 240 + 114 | 270 | 42 - 420 | 269 + 102 | 287.5 | 82 - 420 | 270+ 109 | 292 | 80 - 440 | | 0.277 | |
| Type III/VI/IX | # of daily intakes | 3.25 + 1.41 | 3 | 0 - 6 | 2.58 + 1.52 | 3 | 0 - 6 | 2.47 + 1.44 | 2 | 0 - 6 | | 0.022 | |
|  | Starch Quantity g/day | 108 + 64 | 110 | 25 - 265 | 133 + 76 | 120 | 40 - 385 | 124 + 64 | 120 | 35 - 275 | | 0.167 | |
| Glycosade | # of daily intakes | 4 + 2 | 3 | 1 - 9 | 3.48 + 1.72 | 3 | 1 - 6 | 3.12 + 1.62 | 3 | 1 - 5 | | 0.142 | |
|  | Starch Quantity g/day | 155 + 109 | 122 | 30 - 335 | 180+ 104 | 160 | 40 - 390 | 182 + 109 | 147.5 | 45 - 440 | | 0.263 | |
| UCCS | # of daily intakes | 4 + 2 | 3 | 1 - 9 | 4 + 2 | 3 | 2 - 6 | 3+ 2 | 3 | 1 - 7 | | 0.947 | |
|  | Starch Quantity g/day | 150 + 117 | 120 | 30 - 420 | 203 +132 | 158 | 45 - 420 | 167+115 | 125 | 35 - 390 | | 0.439 | |
| UCCS -> Glycosade | # of daily intakes | 3 + 1 | 3 | 1 - 6 | 2.87 + 0.99 | 3 | 1 - 5 | 2 + 1 | 3 | 1 - 5 | | 0.671 | |
|  | Starch Quantity g/day | 97 + 75 | 75 | 25 - 320 | 123 + 61 | 120 | 40 - 270 | 125 + 64 | 115 | 55 - 270 | | 0.249 | |
| UCCS -> UCCS | # of daily intakes | 4 + 1 | 3 | 2 - 6 | 3.56 + 1.74 | 3 | 1 - 6 | 3 + 2 | 3 | 0 - 6 | | 0.796 | |
|  | Starch Quantity g/day | 149 + 114 | 120 | 45 - 420 | 199 + 143 | 120 | 45 - 420 | 161 + 118 | 125 | 35 - 390 | | 0.521 | |
| Both -> UCCS | # of daily intakes | 5 + 2 | 5 | 3 - 9 | 4 +2 | 4 | 2 - 9 | 4 +2 | 4 | 1 – 9 | | 0.050 | |
|  | Starch Quantity g/day | 215 + 103 | 235 | 40 - 375 | 242 +101 | 250 | 120 - 390 | 248 + 101 | 262.5 | 120 - 440 | | 0.399 | |

**Table 3a Summary of Glycosade intakes for GSD I**

|  | Glycosade intakes GSD Type I | | | | |
| --- | --- | --- | --- | --- | --- |
| Age (years) | Day intake range  g | Median # intakes per day | Median Day time intake  g | Night time intake range g | Median Night time intake g |
| 7 -8 | 50 - 70 | 3 | 70 | 105 | 105 |
| 9 - 10 | 30 - 60 | 3 | 50 | 100 -115 | 100 |
| 11 -14 | 50 - 75 | 4 | 55 | 135 -145 | 140 |
| 15 -17 | 75 | 4 | 75 | 135 - 145 | 140 |
| Adults  (over 18yrs) | 30 - 90 | 4 | 45 | 90 - 165 | 120 |

**Table 3b Summary of Glycosade intakes for ketotic GSD types**

|  | Glycosade intakes GSD Type III, VI & IX | | | | |
| --- | --- | --- | --- | --- | --- |
| Age (years) | Day intake range  g | Median # intakes per day | Median Day time intake  g | Night time intake range g | Median Night time intake g |
| 2 - 4 | 15 - 30 | 2 | 25 | - | - |
| 5 - 6 | 20 - 55 | 2 | 43 | - | - |
| 7 -8 | 20 - 55 | 2 | 43 | - | - |
| 9 - 10 | 20 - 75 | 2 | 30 | 60 - 85 | 65 |
| 11 -14 | 20 - 75 | 1 | 20 | 60 - 90 | 60 |
| 15 -17 | 45 - 60 | 1 | 45 | 60 - 80 | 60 |
| Adults  (over 18yrs) | 60 - 80 | 1 | 60 | 60 - 80 | 68 |

**Table 4: Summary of biochemical parameters**

| GSD Type | Biochemical Parameter | Units | Baseline | | | Visit 4 | | | Visit 5 | | | P value |
| --- | --- | --- | --- | --- | --- | --- | --- | --- | --- | --- | --- | --- |
|  |  |  | mean + SD | median | range | mean + SD | median | range | mean + SD | median | range |  |
| Type Ia | ALT | U/L | 43.82 + 36.27 | 27.5 | 11 - 152 | 37.82 + 31.21 | 24.5 | 13 - 126 | 32.65 + 23.35 | 24 | 12-98 | 0.299 |
|  | AST | U/L | 40.13 + 20.25 | 35 | 21 - 105 | 43.67+ 37.17 | 28 | 19 - 178 | 38.95 + 26.26 | 28 | 15 - 113 | 0.845 |
|  | Triglycerides | mmol/L | 4.31 + 2.7 | 3.48 | 1.44 - 12.7 | 3.87 + 2.21 | 3.02 | 1.06 - 9.46 | 4.32 + 2.78 | 3.42 | 1.34 - 13.4 | 0.72 |
|  | HDL Cholesterol | mmol/L | 0.95 +0.24 | 0.92 | 0.4 - 1.37 | 0.97 + 0.18 | 0.98 | 0.5 - 1.22 | 0.93 + 0.21 | 0.95 | 0.3 - 1.22 | 0.945 |
|  | LDL Cholesterol | mmol/L | 2.63 + 1.27 | 2.54 | 0.04 - 4.8 | 3 ++ 1.18 | 2.59 | 0.6 - 5.8 | 2.58 + 1.15 | 2.3 | 0.2 - 5.7 | 0.642 |
|  | Total Cholesterol | mmol/L | 5.32 +1.33 | 5.3 | 1.8 - 7.7 | 5.64 + 1.61 | 5.4 | 2.6 - 9.5 | 5.15 + 1.24 | 5.2 | 2.4 - 7.3 | 0.795 |
| Type III/VI/IX | Protein | g/L | 70.42 + 2.94 | 70 | 66 - 78 | 72.14 + 4.43 | 72 | 62 - 84 | 72.86 + 4.25 | 73 | 65 - 85 | 0.09 |
|  | ALT | U/L | 151.28 + 175.49 | 88 | 13 - 687 | 113.07 + 124.33 | 54 | 14 - 434 | 132.36 + 150.71 | 71 | 14 - 673 | 0.428 |
|  | AST | U/L | 119.71 + 135.14 | 72 | 21 - 592 | 84.62 + 86.66 | 56.5 | 18 - 309 | 111.63 + 119.54 | 82 | 13 - 493 | 0.453 |
|  | Triglycerides | mmol/L | 1.82 + 0.97 | 1.71 | 0.53 - 5.01 | 1.81 + 1.55 | 1.48 | 0.52 - 8.9 | 2 + 2.02 | 1.59 | 0.52 - 11.2 | 0.791 |
|  | HDL Cholesterol | mmol/L | 0.93 + 0.33 | 0.9 | 0.27 - 1.63 | 0.99 ++ 0.33 | 1 | 0.39 - 1.89 | 1.05 + 0.3 | 1.01 | 0.31 - 1.76 | 0.208 |
|  | LDL Cholesterol | mmol/L | 2.54 + 1.15 | 2.52 | 0.06 - 5.31 | 2.56 + 0.8 | 2.57 | 1.12 - 4.7 | 2.56 + 0.77 | 2.48 | 1.4 - 3.9 | 0.933 |
|  | Total Cholesterol | mmol/L | 4.14 + 1.09 | 4.1 | 2.4 - 6.7 | 4.25 + 0.95 | 4.1 | 2.9 - 7 | 4.42 + 1.23 | 4.3 | 3 - 9.2 | 0.437 |

ALT Alanine transaminase, AST Aspartate aminotransferase

## Figures

Figure 1: Consort Diagram


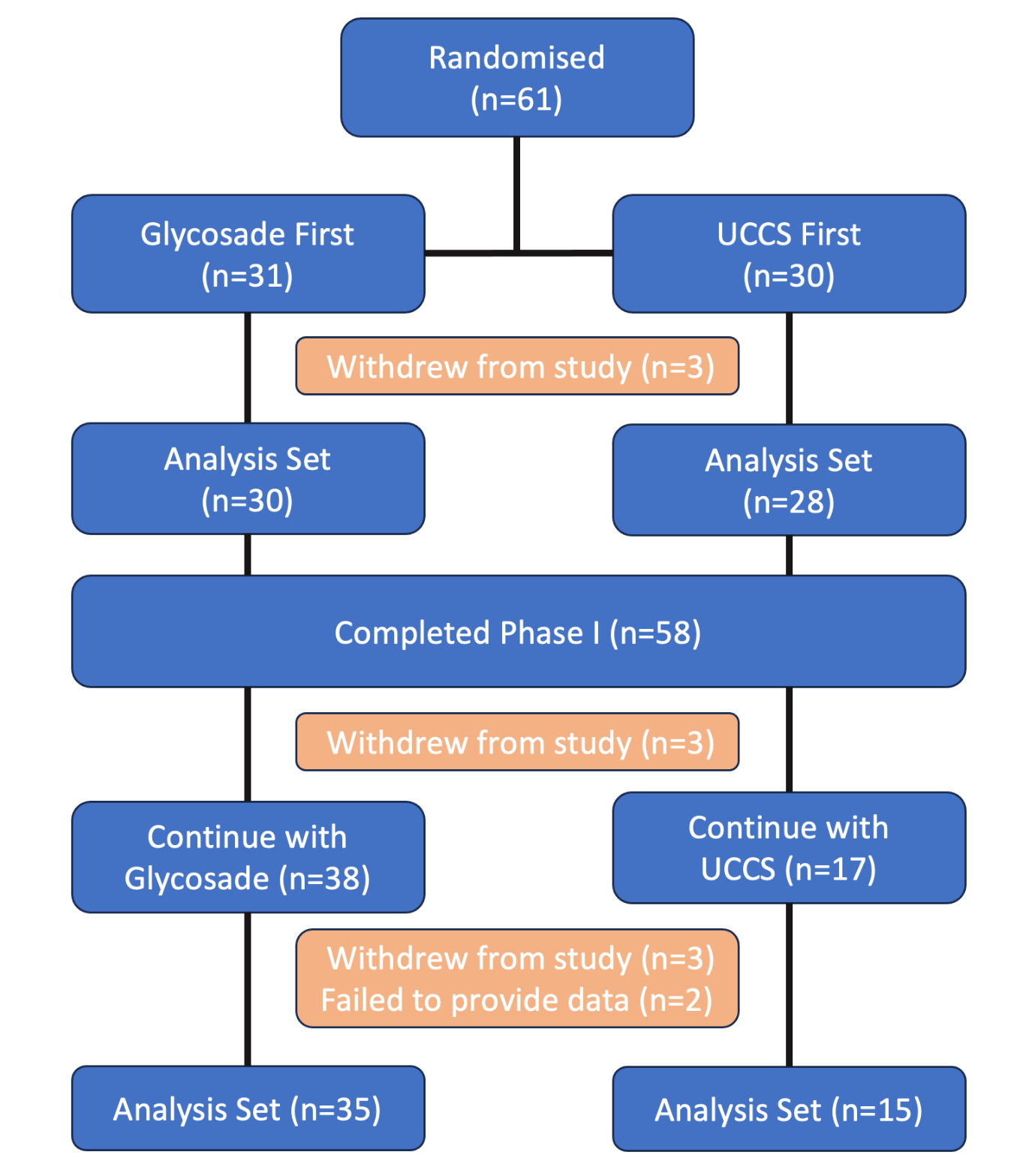


Figure 2: Bar plot to show the number of participants selecting Glycosade or UCCS
